# *Trichoderma* enriches *Burkholderia* via cross-feeding of degradation intermediates to enhance atrazine degradation and alleviate soybean phytotoxicity

**DOI:** 10.1093/ismejo/wrag152

**Published:** 2026-06-29

**Authors:** Haonan Jiang, Manman Zhang, Raja Asad Ali Khan, Jing Zhao, Jumei Hou, Tong Liu

**Affiliations:** Sanya Institute of Breeding and Multiplication, Engineering Center of Agricultural Microbial Preparation Research and Development of Hainan/School of Tropical Agriculture and Forestry, Hainan University, Haikou 570228, China; Sanya Institute of Breeding and Multiplication, Engineering Center of Agricultural Microbial Preparation Research and Development of Hainan/School of Tropical Agriculture and Forestry, Hainan University, Haikou 570228, China; School of Life and Health Sciences, Hainan University, Haikou 570228, China; Sanya Institute of Breeding and Multiplication, Engineering Center of Agricultural Microbial Preparation Research and Development of Hainan/School of Tropical Agriculture and Forestry, Hainan University, Haikou 570228, China; Sanya Institute of Breeding and Multiplication, Engineering Center of Agricultural Microbial Preparation Research and Development of Hainan/School of Tropical Agriculture and Forestry, Hainan University, Haikou 570228, China; Sanya Institute of Breeding and Multiplication, Engineering Center of Agricultural Microbial Preparation Research and Development of Hainan/School of Tropical Agriculture and Forestry, Hainan University, Haikou 570228, China; Sanya Institute of Breeding and Multiplication, Engineering Center of Agricultural Microbial Preparation Research and Development of Hainan/School of Tropical Agriculture and Forestry, Hainan University, Haikou 570228, China

**Keywords:** atrazine, *Trichoderma*, rhizosphere microbiota, microbial community

## Abstract

The widespread agricultural use of atrazine threatens soil health, and residual phytotoxicity in corn-soybean rotation systems necessitates sustainable remediation strategies. By leveraging the atrazine-degrading fungus *Trichoderma lentiforme* HN154, we achieved an 80.3% removal of atrazine (500 mg/kg) in non-sterilized soils from a corn planting system within 14 days, 22.1% higher degradation than in sterilized soil, while concurrently alleviating phytotoxic symptoms in soybean plants. Metagenomic analysis revealed that colonization by *T. lentiforme* HN154 drove restructuring of microbial networks, enriching the keystone family Burkholderiaceae, which was strongly associated with atrazine catabolism and four key catabolic enzymes [EC 3.5.4.43 (*atzB*), EC 3.5.1.131 (*atzE*), EC 3.5.1.54 (*atzF*), EC 3.5.4.42 (*atzC*)]. Among 23 rhizosphere isolates, the *Burkholderia* strains Bur-4, Bur-5, and Bur-14 showed the highest atrazine degradation rates (26.3% to 29.4%) within 72 h. A *Trichoderma*–*Burkholderia* synthetic consortium further enhanced remediation by boosting plant antioxidant defenses (SOD, POD, CAT) and reducing oxidative damage (MDA). Mechanistically, intermediates (hydroxyatrazine and biuret) generated during *T. lentiforme* HN154-mediated degradation stimulated *Burkholderia* chemotaxis, swarming, and swimming motility, while cross-feeding on these metabolites synergistically accelerated bioaugmentation (the *Trichoderma*–*Burkholderia* synthetic consortium achieved rapid atrazine degradation of 86.3% within 168 h). This study reveals tripartite interactions among exogenous microbial degraders, pollutant metabolites, and indigenous microbiota, offering a strategic foundation for microbiome-guided, precision bioaugmentation to restore soil ecological health and crop resilience.

## Background

Atrazine (2-chloro-4-ethylamino-6-isopropylamino-1,3,5-triazine) (Atz) is a herbicide that inhibits photosystem II (PSII) in chloroplasts, leading to plant chlorosis and withering. As one of the most effective and widely used herbicides in modern agriculture for controlling annual grasses and broadleaf weeds [1, 2], atrazine is the second most extensively applied herbicide globally [3]. It is predominantly used in corn, pineapple, sorghum, and sugarcane production systems. Residual atrazine in agricultural soils can reach as high as 1100 mg/kg, posing severe phytotoxicity to crops such as soybean (*Glycine max*), sorghum, oats, wheat, and sweet potatoes [4]. Beyond its agronomic impacts, atrazine functions as a potent endocrine disruptor with significant teratogenicity and carcinogenicity. It damages mitochondria and other organelles in mammalian cells, including those in humans, contributing to neurological impairments, dysregulation of the endocrine and immune systems, and reproductive cancers [5]. These findings underscore the urgent need for effective remediation strategies to mitigate the environmental and health risks associated with atrazine.

Approaches to eliminate atrazine residues include physical degradation [6], chemical degradation [7], photolysis [8], and bioremediation. However, physical and chemical methods often face limitations in large-scale applicability due to being time-consuming, labor-intensive, and inefficient, as well as posing risks of secondary soil contamination. In contrast, bioremediation, specifically biodegradation for detoxification, is recognized as a sustainable, eco-friendly, and highly promising strategy for atrazine-contaminated soils [9]. Since the discovery of the first atrazine-degrading microbial strain, *Pseudomonas* sp. ADP [10], numerous microbial degraders have been identified, including *Pseudomonas aeruginosa* [11], *Nocardioides* sp. SP12 [12], and others. Bioaugmentation, a key bioremediation strategy involving the introduction of exogenous microbial degraders such as *Rhodococcus* sp., *Acinetobacter* sp., and *Bacillus* sp. [13], has been widely explored. However, the success of bioaugmentation depends on the survival and activity of these degraders in competitive soil ecosystems. To tackle these challenges, many studies have tried to enhance the adaptability of microbial degraders by supplementing organic amendments like pig manure and rice straw to improve survival and degradation capacity [14]. Conversely, a study has reported that biochar derived from organic materials may delay the degradation of atrazine [15]. Therefore, it is essential to investigate alternative strategies to enhance the functional stability of microorganisms employed in biodegradation processes.

Indigenous soil microbial communities also play pivotal roles in bioremediation processes, directly influencing the productivity and stability of ecosystems [16]. Shifts in their composition are widely recognized as sensitive indicators of soil quality, health status, and ecological dynamics [17]. Studies have demonstrated that atrazine exerts toxic effects on soil microbiota and that, even at trace concentrations, it significantly alters microbial diversity and functional profiles [18]. The application of degradative bacterial strains, such as *Arthrobacter* sp. HB-5 [19], *Paenarthrobacter* sp. AT-5 [20], and *Chenggangzhangella methanolivorans* CHL1, can partially restore bacterial diversity that was reduced by atrazine residues.

In the rhizosphere ecosystem, plants and microbes often establish reciprocal benefits: microbial degradation of atrazine and other pollutants enhances plant growth, while plants supply nutrients and energy to sustain microbial communities [21]. This mutualism promotes soil microbial diversity and richness [22], fostering opportunities for the coexistence of both exogenous microbial degraders and indigenous cooperative strains. However, the tripartite interactions among atrazine residues as soil pollutants, exogenous microbial degraders, and indigenous microbial communities remain poorly understood. Addressing this knowledge gap through systematic elucidation of their interplay could help identify indigenous microbial taxa that synergistically enhance exogenous degraders, offering a strategic approach to stabilize bioaugmentation processes by leveraging indigenous functional synergies.

*Trichoderma* sp., a ubiquitous fungal genus across diverse ecosystems, has the capacity to produce enzymes capable of degrading xenobiotic pollutants in soil [23] and exhibits remarkable efficacy in detoxifying recalcitrant organochlorine pesticides, including highly toxic and persistent compounds such as DDT, dieldrin, and endosulfan [24]. However, the ecological and mechanistic consequences of introducing exogenous *Trichoderma* fungi as biodegraders into complex soil microbiomes remain poorly understood. Key unresolved questions include: (i) How do indigenous microbial communities modulate the degradation efficiency of bioaugmented *Trichoderma* strains? And (ii) which specific indigenous microbial interactions govern the degradation of pollutants like atrazine and alleviate associated phytotoxicity? To address these questions, we used *Trichoderma lentiforme* HN154 as a microbial degrader and employed metagenomic profiling, culturomics analyses, and synthetic community construction to investigate how restructuring of the microbial community and changes in functional gene abundance govern the cooperative degradation of atrazine. We further addressed the foundational challenge of unraveling tripartite interactions among fungal inoculants, xenobiotic contaminants, and indigenous microbiota (e.g. *Burkholderia*) in the soybean rhizosphere through in vitro interaction assays. Our results suggest that chemotaxis-mediated enrichment and metabolic cross-feeding are key mechanisms for establishing a stable and cooperative microbial network. This advances the rational design of microbial consortia for bioaugmentation, with the dual objectives of accelerating contaminant removal and fostering resilience to functionally restore soil ecosystems.

## Materials and methods

### Soil preparation and experimental design

The soil was collected from the root zone (20–30 cm depth) of maize plants at the Xinnongke Experimental Base (19°51′ N, 109°49′ E) of Hainan University, Danzhou, Hainan Province, China [25]. This site has a long-term maize cultivation history with no prior atrazine application. The soil was air-dried indoors and sieved through a 100-mesh sieve. A portion of the dried soil was sterilized by autoclaving at 121°C for 30 min, while the rest remained non-sterilized [26]. Both soil samples were spiked with an atrazine-acetone solution (atrazine analytical standard (purity ≥98%), acetone (pesticide grade), Sigma-Aldrich, St. Louis, MO, USA) to achieve a final atrazine concentration of 500 mg/kg (on a dry weight basis). To remove residual acetone and eliminate any solvent effects on the indigenous microbiota, samples were incubated in a fume hood for 24 h (protected from light) [27, 28]. This process yielded the final sterilized and non-sterilized atrazine-contaminated soils for use in the pot experiments. The *T. lentiforme* HN154 strain was isolated and preserved by the Key Laboratory of Green Prevention and Control of Tropical Plant Diseases and Pest (Ministry of Education). When exposed to a high atrazine concentration (500 mg/l), HN154 strain achieved a degradation efficiency of 90.1% over seven days in a controlled laboratory setting [29]. To evaluate the role of indigenous microbiota in *T. lentiforme* HN154-mediated mitigation of soybean phytotoxicity, a factorial pot experiment was established with four treatments: (i) AtrSs: Sterilized atrazine-contaminated soil (500 mg/kg). (ii) AtrSs.HN154: Sterilized atrazine-contaminated soil +50 ml *T. lentiforme* HN154 spore suspension (10^8^ CFU/ml). (iii) AtrUs: Non-sterilized atrazine-contaminated soil (500 mg/kg). (iv) AtrUs.HN154: Non-sterilized atrazine-contaminated soil +50 ml *T. lentiforme* HN154 spore suspension (10^8^ CFU/ml). The *T. lentiforme* HN154 strain was first activated and cultured on Potato Dextrose Agar (PDA, BD Difco, US) at 28°C, with subculturing performed every 5 days. After three successive subculturing cycles, spores were harvested from the plates by washing with sterile water containing 0.05% Tween 80 (Sigma-Aldrich). This spore suspension was then adjusted with sterile water to a final concentration of 10^8^ CFU/ml [30].

Soybean seeds (Shandong Shouguang Xinran Horticulture Co., Ltd, Shandong, China) were surface-sterilized by sequential immersion in 70% (v/v) ethanol for 1 min and 1% (w/v) sodium hypochlorite for 10 min, followed by five rinses with sterile distilled water. After sterilization, seeds were placed on sterile filter paper moistened with sterile water and germinated at 30 ± 1°C for 48 h. Uniformly germinated seedlings were transplanted into pots (11 cm × 9.5 cm × 8 cm, 500 g soil per pot) at a density of four seedlings per pot. All treatments were maintained under controlled conditions: 27°C/20°C, day/night cycle (12 h/12 h), and 60% soil moisture. The experiment was repeated five times independently, and each treatment comprised five pots each time. After 14 days of cultivation, plant height, main root length, and fresh weight per plant were measured. Plant height was measured with a ruler from the cotyledon node to the peak of the main stem. The intact root system was excavated, gently rinsed with sterile water to remove soil, and blotted dry. The root system was laid flat, and the primary root length was measured from the base to the tip along the main root using a ruler. The fresh weight of the soybean plants was determined using an analytical balance [31, 32].

### Soil sampling and atrazine degradation analysis

The rhizosphere samples were collected around soybean roots using a elution method with phosphate-buffered saline (PBS, Solarbio Science & Technology Co., Ltd, Beijing, China) [32]. In the aforementioned pot experiment with four treatments (AtrSs, AtrSs.HN154, AtrUs, and AtrUs.HN154), five soybean plants were randomly selected from each treatment, and their rhizosphere soils were pooled together. This procedure was repeated four times, generating 16 soil samples (4 treatments × 4 biological replicates = 16 rhizosphere samples). All samples were stored at −80°C and divided into two subsamples: one for DNA extraction (excluding the AtrSs treatment) and the other for quantifying residual atrazine.

The quantification method for residual atrazine was optimized based on the approach established [21, 33]. For atrazine extraction, 5 g of rhizosphere soil was subjected to sequential extraction with 5 ml dichloromethane (HPLC-grade, Sigma-Aldrich) (2–3 cycles). For hydroxyatrazine (≥98%, Sigma-Aldrich), extraction was performed using an acidic solvent mixture of 0.1 M HCl and acetonitrile. The extraction of biuret (≥99%, Supelco, Bellefonte, PA, USA) involves dissolution in methanol at 65°C with stirring for 2–6 h. The combined extracts were concentrated via rotary evaporation (Büchi R-300, Büchi Labortechnik, Flawil, Switzerland) water bath (temperature ≤30°C), reconstituted in 5 ml HPLC-grade methanol, and filtered through a 20-μm membrane (Millex polyethersulfone, Millipore, Burlington, MA, USA). Atrazine quantification was performed using an Agilent 1260 HPLC system (Agilent Technologies, Santa Clara, CA, USA) equipped with an Eclipse XDB-C18 column (250 mm × 4.6 mm, 5 μm particle size). The analytical method was calibrated according to a previously published study [34]. The following final procedure was employed for the atrazine detection process: the mobile phase was a 50:50 (v/v) mixture of water and methanol, the flow rate was 1 ml/min, with detection wavelength at 220, injection volume, column temperature, and run time were set to 20 μl, 30°C, and 30 min, respectively. Peak area versus atrazine concentration yielded the calibration curve (Supplementary Figs 1 and 2). Soil extracts above the calibrated limit (10 mg/l) were diluted 10-fold in HPLC-grade methanol and re-injected, those >100 mg/l were diluted 100-fold. Final concentrations were calculated from the instrumental response multiplied by the respective dilution factor, permitting accurate quantification to 600 mg/l without system modification. All recovery, limit of detection, retention time, and other validation data for the extended range are given in Supplementary Table 1. The degradation rate is expressed as the percentage of atrazine removed relative to the initial concentration.

The following HPLC procedures were employed for the analysis: for hydroxyatrazine, a gradient elution with 0.1 M phosphate buffer (pH 3.5, Sigma-Aldrich) and acetonitrile was used, with detection at 254 nm. For biuret, an isocratic elution with 10 mM ammonium acetate buffer (pH 6.5) and methanol (90:10, v/v) was used, with detection at 200 nm. For both hydroxyatrazine and biuret, the flow rate was 1 ml/min, and the injection volume, column temperature, and run time were set to 20 μl, 30°C, and 10 min, respectively. External calibration curves (peak area vs. concentration) were established for hydroxyatrazine and biuret (Supplementary Figs 3–5). All analyses included five independent replicates.

### Metagenomic sequencing and bioinformatics analysis

DNA extraction and high-throughput sequencing were performed by Novogene Co., Ltd. (Beijing, China). Total DNA was extracted from 0.25 g (fresh weight) rhizosphere soil using the DNeasy PowerSoil Kit (Qiagen, Hilden, Germany) following the manufacturer’s protocol with one modification: bead-beating was performed at 6 ms^−1^ for 45 s in a FastPrep-24 [35]. DNA quantity and integrity were assessed on a NanoDrop-2000 and by 1% (w/v) agarose-gel electrophoresis, respectively. Qualified DNA samples (effective concentration > 3 nM) were randomly sheared into short fragments, end-repaired, A-tailed, ligated to Illumina adapters, PCR-amplified, size-selected, and purified to generate sequencing libraries. Library concentration was determined with Qubit and qPCR, and size distribution was verified on an Agilent 2100 Bioanalyzer. Libraries were pooled and sequenced on a NovaSeq 6000 System (Illumina) with PE150 cycles [36].

Raw reads were subjected to quality control and host DNA removal using Readfq (https://github.com/cjfields/readfq) and Bowtie2 (parameters: -m 200, -x 400, identity ≥95%) [37], yielding high-quality clean reads. De novo assembly of clean reads was conducted with MEGAHIT (v1.2.9) using the meta-large preset (presets meta-large) [38], and scaftigs (≥500 bp) were obtained by breaking scaffolds at N junctions [39]. Open reading frames (ORFs) were predicted using MetaGeneMark (v3.38) for scaftigs ≥500 bp, and sequences <100 nt were discarded [40]. ORFs were de-replicated at 95% identity/90% coverage using CD-HIT (v4.8.1) (-c 0.95, -G 0, -aS 0.9, -g 1, -d 0) to generate the non-redundant gene catalog [41].

Clean reads were aligned to the catalog with Bowtie2 (-end -to -end, -sensitive, -I 200, -X 400) [42], genes with ≤2 mapped reads were removed to yield Unigenes [43], and gene abundance (reads per kilobase, RPKM) was calculated as previously described [44]. The standard RPKM formula was calculated as follows: for sample i, given r (reads mapped to gene k) and L (gene length in kilobases).


$$ {G}_k=\frac{r_k}{L_k}.\frac{1}{\sum_{i=1}^n\frac{r_i}{L_i}} $$

Detailed read counts and library sizes are provided in the Supplementary Data 1. Taxonomic annotation was performed by aligning Unigenes to NCBI-NR using DIAMOND (blastp, e-value ≤10^−5^) followed by LCA algorithm assignment [45], and functional annotation against Kyoto Encyclopedia of Genes and Genomes (KEGG) databases was conducted using DIAMOND with the best-hit strategy (BLASTP, e-value ≤10^−5^) [46]. Antibiotic-resistance genes were identified by mapping Unigenes to the CARD database using RGI (built-in blastp, e-value <10^−30^) [47].

For microbiome community comparisons, PERMANOVA (Bray–Curtis distance, 999 permutations) was performed, multivariate dispersion homogeneity (PERMDISP) was confirmed (*P* > .05) and reported with pseudo-*F*, df, *R*^2^, and exact *P* values [48]. LEfSe analysis was used to identify differentially enriched biomarkers (i.e. metagenomic analyses identify differentially abundant microbes as biomarkers) (LDA score > 4.0, *P* < .05) [49]. Welch’s *t-*test with Storey false discovery rate (FDR) correction (*q* < 0.05) was applied for family-level comparisons, and the results were visualized as amplification histogramsin STAMP (v2.0.0) [50].

### Isolation and screening of atrazine-degrading bacteria in soil

Culturable bacteria were isolated through serial dilution plating from the rhizosphere soil of soybean plants in AtrUs.HN154 treatment, using inorganic salt medium (ISM, Burk medium removed carbon and nitrogen sources) [51] supplemented with 500 mg/l atrazine as the sole carbon/nitrogen source to obtain single colonies. The colonies were repeatedly purified via streak plate method on PDA agar (three successive passages, each with 5 days at 30°C). Purified colonies were taxonomically identified by amplifying 16S rRNA genes using primers 27F (5′-AGAGTTTGATCCTGGCTCAG-3′) and 1492R (5′-GGTTACCTTGTTACGACTT-3′) [52]. For degradation screening, bacterial suspensions (10 μl, OD600 nm = 1.0) from 72-h PDA broth cultures were inoculated into 50 ml of fresh ISM medium containing 500 mg/l of atrazine and incubated at 28°C with 180 rpm agitation for 72 h. The obtained suspension was then subjected to liquid–liquid extraction with an equal volume of dichloromethane (DCM, HPLC-grade, Sigma-Aldrich) through vigorous shaking for 30 min. After phase separation, the organic layer (DCM phase) was carefully transferred to a fresh centrifuge tube. This extraction procedure was repeated three times, and the combined organic extracts were dehydrated with 1 g anhydrous MgSO_4_. The desiccated extract was concentrated via rotary evaporation water bath (temperature controlled at ≤30°C) and reconstituted in HPLC-grade anhydrous methanol. Prior to analysis, the solution was filtered through 0.22 μm nylon membrane filters. Atrazine residues were quantified using HPLC under the aforementioned chromatographic conditions [53].

### Synthetic consortium assembly and phytotoxicity assessment

The synthetic consortium was established using *T. lentiforme* HN154 as the foundational strain. *Burkholderia* strains Bur-4, Bur-5, and Bur-14 were individually purified by streaking. Single colonies of each strain were selected and pre-cultured in Potato Dextrose (PD, BD Difco, US) broth with shaking. The synthetic bacterial community Burk was established by mixing equal volumes of three indigenous *Burkholderia* strains, Bur-4, Bur-5, and Bur-14, each adjusted to equal cell densities of 1 × 10^8^ CFU/ml, to constitute a synthetic bacterial community, designated Burk. This bacterial community Burk was then blended with *T. lentiforme* HN154 spore suspension (1 × 10^8^ CFU/ml) at a 1:1 volumetric ratio, yielding the synthetic consortium HN154 + Burk (final cell density: 1 × 10^8^ cells/ml). A pot experiment with five treatments was conducted to evaluate the consortium’s capacity to alleviate atrazine-induced soybean stress. Each 11 cm (diameter) × 9.5 cm (height) pot containing 500 g of processed soil was assigned to the following groups: (i) Control: Sterile soil without atrazine, (ii) AtrSs: Sterile soil spiked with 500 mg/kg atrazine, (iii) AtrSs.HN154: AtrSs +50 ml HN154 suspension (1 × 10^8^ CFU/ml), (iv) AtrSs.Burk: AtrSs +50 ml bacterial community Burk (1 × 10^8^ CFU/ml), (v) AtrSs.HN154.Burk: AtrSs +50 ml consortium HN154 + Burk (1 × 10^8^ cells/ml). Soybean seeds were germinated, transplanted, and cultivated following the methods described above. Soybean agronomic traits, including plant height, fresh weight, and root length, were measured, and rhizosphere atrazine residues were quantified after 14 days [34].

To systematically assess the consortium’s phytotoxicity efficacy, three hallmark antioxidant enzymes in soybean roots were analyzed: superoxide dismutase (SOD, EC 1.15.1.1), peroxidase (POD, EC 1.11.1.7), and catalase (CAT, EC 1.11.1.6). Concurrently, malondialdehyde (MDA) levels were determined as a lipid peroxidation biomarker [54]. All analyses were performed with five biological replicates per treatment group. Superoxide dismutase (SOD, Enzyme Commission 1.15.1.1), peroxidase (POD, EC 1.11.1.7), and catalase (CAT, EC 1.11.1.6) activities, as well as malondialdehyde (MDA) content (SOD kit cat. 19 160, POD kit cat. P8126, CAT kit cat. CAT100, MDA kit cat. MAK085, all Sigma-Aldrich), were determined with corresponding assay kits according to the manufacturer’s instructions. Fresh root tissue (0.1 g) was homogenized in ice-cold 50 mM potassium-phosphate buffer (pH 7.8) containing 1% (w/v) polyvinylpolypyrrolidone (PVPP, Sigma-Aldrich) and centrifuged at 12 000 × *g* for 15 min at 4°C. The resulting supernatant was used directly for colorimetric measurements on a Synergy H1 microplate reader (BioTek Instruments, Winooski, VT, USA) at 450 nm (SOD), 470 nm (POD), 240 nm (CAT), and 532 nm (MDA). Enzyme activities are expressed as U mg^−1^ protein, and MDA as nmol g^−1^ fresh weight. Protein concentration was determined by the Bradford method (Bradford reagent, Sigma-Aldrich) using bovine serum albumin as standard [55].

### Effects of intermediates in atrazine degradation on in vitro growth characteristics of core bacterial

To evaluate the effects of *T. lentiforme* HN154-mediated atrazine degradation on colonization traits of the Burk community, *T. lentiforme* HN154 was cultured in ISM containing 500 mg/l atrazine as the sole carbon/nitrogen source at 28°C for 3 days. Fungal cells were removed by centrifugation (4000 g, 10 min), and the supernatant was filtered through a 0.22 μm nylon syringe filter. The filtrate was split into two aliquots: one for extraction, rotary evaporation, and HPLC identification of degradation intermediates (hydroxyatrazine, biuret), the other retained for downstream bioassays.

Chemotaxis was quantified using a modified capillary assay [56]. The apparatus consisted of a 200 μl pipette-tip chamber containing 100 μl of *Burkholderia* suspension (OD_600_ nm = 0.1, overnight culture in ISM). A 4-cm 25-gauge needle (Becton-Dickinson, Franklin Lakes, NJ, USA) connected to a 1 ml tuberculin syringe (Becton–Dickinson) served as the chemotaxis capillary and was loaded with 200 μl of test solution. After 2 h incubation at room temperature, the contents of the needle were expelled, serially diluted, and plated on ISM agar. Colonies were counted after 48 h at 28°C.

The relative chemotaxis index (*Rc*) was calculated as the ratio of CFUs (Colony-Forming Units) in the test capillary to those in the control capillary; *Rc* of 2 or greater is considered significant for this method. Each assay was performed in triplicate.

The swimming and swarming assay was performed as previously described [57] with modifications. Briefly, 5 μl of bacterial suspension (OD_600_ nm = 1.0) was spot-inoculated onto synthetic minimal medium (SMM) prepared for swimming (0.3% agar) or swarming (0.8% agar), supplemented with 50 mg/l of the test solution. Plates were incubated upright at 28°C for 24 h, and colony diameters were measured. The swimming/swarming index (*Rs*) was calculated as (diameter test-diameter control)/diameter control, *Rs* of 2 or greater is considered significant for this method. All motility assays were run in triplicate with sterile ISM (an equivalent volume of solvent was substituted for the test solution) as the negative control.

Test solution conditions: ISM (inorganic salt medium). CK (*Burkholderia* sp. cultured in sterile ultrapure water). AISM (*Burkholderia* sp. cultured in ISM with 50 mg/l atrazine as the sole carbon and nitrogen source). HISM (*Burkholderia* sp. cultured in ISM with 50 mg/l hydroxyatrazine as the sole carbon and nitrogen source). BISM (*Burkholderia* sp. cultured in ISM with 50 mg/l biuret as the sole carbon and nitrogen source). BHISM (*Burkholderia* sp. cultured in ISM with 50 mg/l biuret and hydroxyatrazine as dual carbon/nitrogen sources). HCB (*Burkholderia* sp. cultured in cell-free broth prepared by growing HN154 in ISM for 72 h and then removing the fungal cells). AHCB (*Burkholderia* sp. cultured in HN154-culture broth-i.e, atrazine-based ISM precultured with HN154 for 72 h and then freed of the fungus, SMM based on atrazine after HN154 culture for 72 h and subsequent removal of HN154). Atz (*Burkholderia* sp. cultured in SMM supplemented with 50 mg/l atrazine). Hyd (*Burkholderia* sp. cultured in SMM supplemented with 50 mg/l hydroxyatrazine). Biu (*Burkholderia* sp. cultured in SMM supplemented with 50 mg/l biuret). Hyd-Biu (*Burkholderia* sp. cultured in SMM supplemented with both 50 mg/l hydroxyatrazine and biuret). All assays used the culture of sterile ultrapure water as a control, and were conducted with three replicates.

### Statistical analysis

All data were analyzed in SPSS 24.0 (IBM Corp., USA). One-way ANOVA followed by Tukey’s HSD post hoc test was used to evaluate differences among multiple groups, independent-samples *t-*test was applied for pairwise comparisons. Significance was set at *P* < .05. Data are presented as means ± standard deviation (SD) with exact sample sizes (*n*) given in each figure legend. Normality (Shapiro–Wilk) and homogeneity of variances (Levene) were verified prior to parametric tests. When assumptions were violated, data were log-transformed or analyzed using Welch’s ANOVA followed by Games–Howell post hoc comparisons. Correlations were assessed with Spearman’s rank correlation (robust to non-normality); linear relationships were evaluated by simple linear regression. For multiple comparisons, two-sided Welch’s *t*-tests (unequal variances) with 99% confidence intervals constructed, multiple testing was controlled using the Storey FDR approach. Differential biomarkers between AtrUs and AtrUs.HN154 were identified by LEfSe (LDA > 4, *P* < .05, Kruskal–Wallis test with Storey FDR correction).

## Results

### *Trichoderma lentiforme* HN154 degrades atrazine to mitigate its phytotoxicity in soil environments

To evaluate the atrazine degradation efficiency and phytotoxicity mitigation potential of *T. lentiforme* HN154 in sterilized or non-sterilized soil, a four-treatment pot experiment was conducted (Fig. 1A). *Trichoderma lentiforme* HN154, as the primary degrader, significantly alleviated soybean phytotoxicity and enhanced atrazine degradation (Fig. 1B–E). Soybean plants in the AtrSs group exhibited severe leaf wilting and necrotic lesions, whereas these symptoms were significantly reduced in AtrSs.HN154 and AtrUs.HN154 groups. Compared to AtrSs, the AtrSs.HN154 treatment increased plant height by 11.3%, fresh weight by 28.7%, root length by 48.2% (Fig. 1D), and atrazine degradation efficiency by 211% (58.2% at 168 h) (Fig. 1E). Similarly, relative to AtrUs, the AtrUs.HN154 group showed significant enhancements (*P <* .05) in plant height (20.9%), fresh weight (31.9%), and root length (20.2%), with a 122% increase in atrazine degradation (80.3% at 168 h). The AtrUs.HN154 treatment achieved optimal soybean growth and the highest degradation rate, likely due to synergistic interactions between *T. lentiforme* HN154 and the indigenous microbiome in non-sterilized soil.

**Figure 1:**
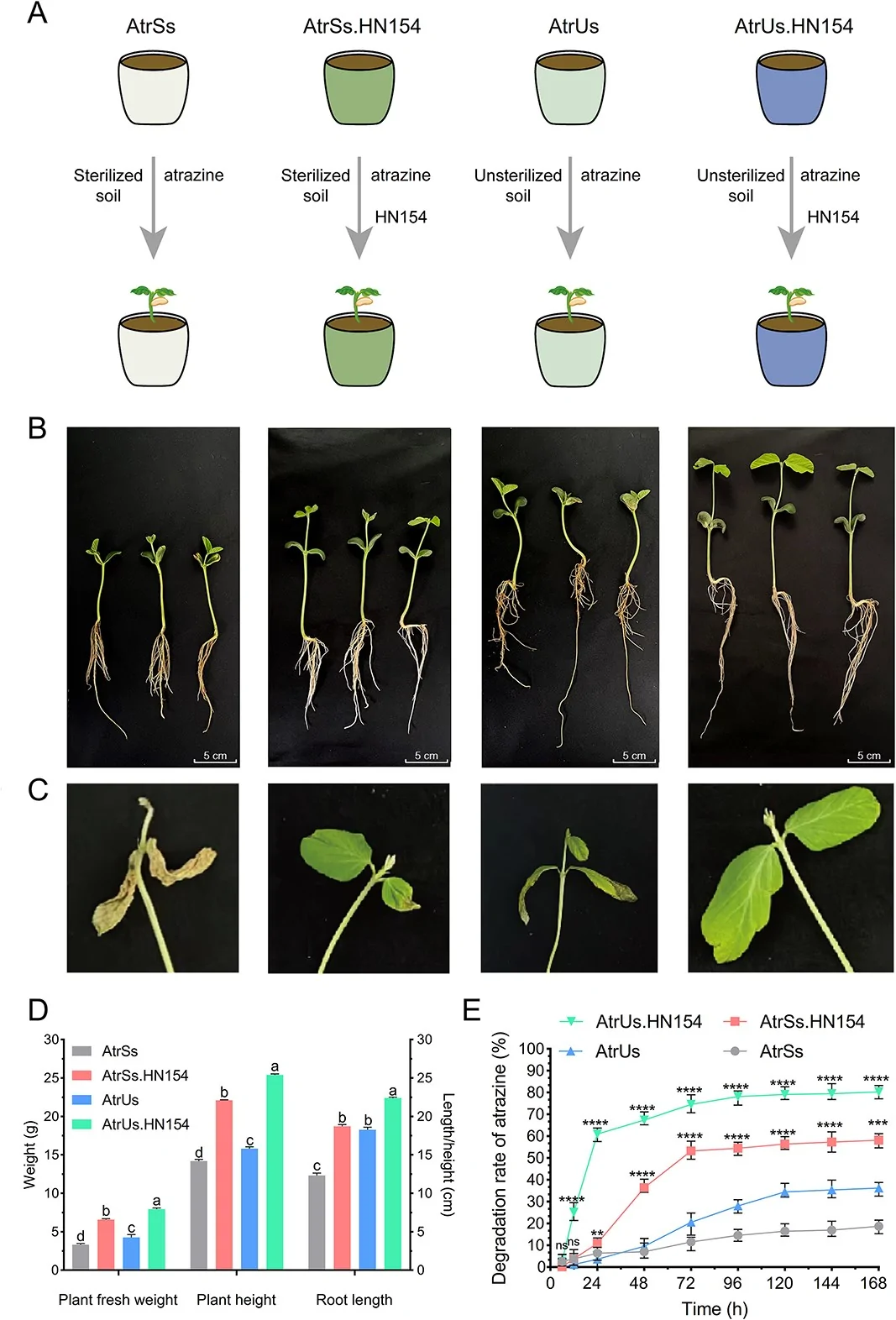
Effects of *T. lentiforme* HN154 on soybean growth and atrazine degradation in sterilized and non-sterilized soil. (A) Schematic diagram of four treatments in pot experiments: AtrSs, AtrSs.HN154, AtrUs, and AtrUs.HN154. (B) Soybean plants at 14 days post-treatment. (C) Leaf morphology at 21 days. (D) Fresh weight, plant height, and root length of soybeans at 14 days. (E) Atrazine degradation rates in soil across time points. Among them, AtrSs: Sterilized atrazine-contaminated soil (500 mg/kg). AtrSs.HN154: Sterilized atrazine-contaminated soil +50 ml HN154 spore suspension (10^8^ CFU/ml). AtrUs: Non-sterilized atrazine-contaminated soil (500 mg/kg). AtrUs.HN154: Non-sterilized atrazine-contaminated soil +50 ml HN154 spore suspension (10^8^ CFU/ml). Data were expressed as means ± SD (*n* = 4). Significant differences among treatments were analyzed by one-way ANOVA followed by Tukey’s HSD test (*P <* .05). In panel D, different lowercase letters denote statistically significant differences. In panel E, asterisks indicate significant differences between AtrUs.HN154 and AtrUs or between AtrSs.HN154 and AtrSs at a specific time point (^***^P < .001, ^****^P < .0001).

### Impact of *T. lentiforme* HN154 on indigenous microbial communities

Metagenomic sequencing of rhizosphere from AtrSs.HN154, AtrUs, and AtrUs.HN154 treatments revealed shifts in microbial composition. A total of 88 bacterial and 15 fungal phyla were identified, with four phyla (Pseudomonadota, Acidobacteriota, Actinomycetota, and *Candidatus* Saccharibacteria) as dominant phyla, constituting 73.3% to 74.9% of relative abundance (Fig. 2A). Principal Coordinates Analysis (PCoA) based on Bray-Curtis distances demonstrated distinct clustering among treatments (*P <* .05), where PCoA1 explained 92.2% of total variance (Fig. 2B, Supplementary Table 2, Supplementary Data 2), indicating that *T. lentiforme* HN154 inoculation was the primary driver of microbial community restructuring.

**Figure 2:**
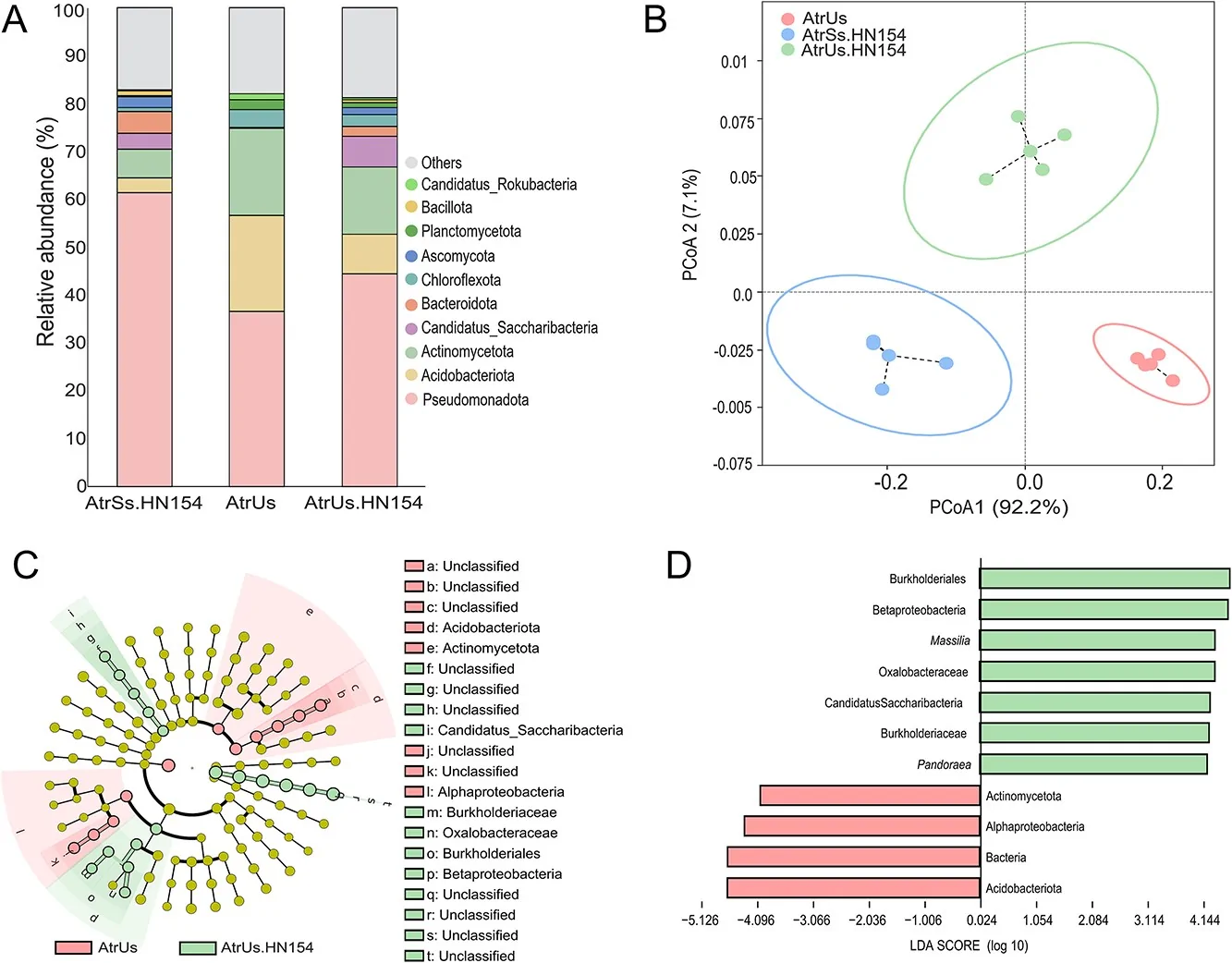
Impact of *T. lentiforme* HN154 on the soybean rhizosphere microbiome under sterilized and non-sterilized soil conditions. (A) Stacked bar plots showing the relative abundance of the top 10 phyla in the rhizosphere microbiome with AtrSs.HN154, AtrUs, and AtrUs.HN154 treatments. (B) Principal coordinates analysis (PCoA) of microbial communities based on Bray–Curtis dissimilarity at the phylum level. (C-D) Biomarker analysis comparing AtrUs and AtrUs.HN154 treatments. (C) Cladogram of differentially enriched taxa identified by LEfSe (linear discriminant analysis effect size): Taxa significantly enriched in AtrUs are shown in red, those enriched in AtrUs.HN154 are in green; yellow indicates taxa not significantly different. (D) Distribution of linear discriminant analysis (LDA) scores for biomarkers differing significantly between treatments (LDA > 4.0, *P* < .05). The bar chart shows species with LDA scores >4. The length of the bar represents the magnitude of the effect of different species. Among them, AtrSs.HN154: Sterilized atrazine-contaminated soil +50 ml *T. lentiforme* HN154 spore suspension (10^8^ CFU/ml). AtrUs: Non-sterilized atrazine-contaminated soil (500 mg/kg). AtrUs.HN154: Non-sterilized atrazine-contaminated soil +50 ml *T. Lentiforme* HN154 spore suspension (10^8^ CFU/ml). Data are presented as mean ± SD from four independent experiments (*n* = 4).

### Rhizosphere biomarkers responsive to *T. lentiforme* HN154

To identify indigenous microbial biomarkers in non-sterilized soil responsive to *T. lentiforme* HN154, LEfSe (LDA Effect Size) analysis was performed between AtrUs and AtrUs.HN154 treatments (Fig. 2C and D, Supplementary Tables 3 and 4). The biomarkers in the AtrUs group included Actinomycetota, Alphaproteobacteria, and Acidobacteriota, whereas AtrUs.HN154 biomarkers comprised Candidatus Saccharibacteria, Betaproteobacteria, Burkholderiales, Burkholderiaceae, Oxalobacteraceae, *Massilia*, and *Pandoraea*. Among these, Burkholderiales exhibited the highest LDA score (log10 > 4.0) and was significantly enriched in AtrUs.HN154 (Fig. 3A, Supplementary Data 3), served as the most discriminative biomarker between treatments.

**Figure 3:**
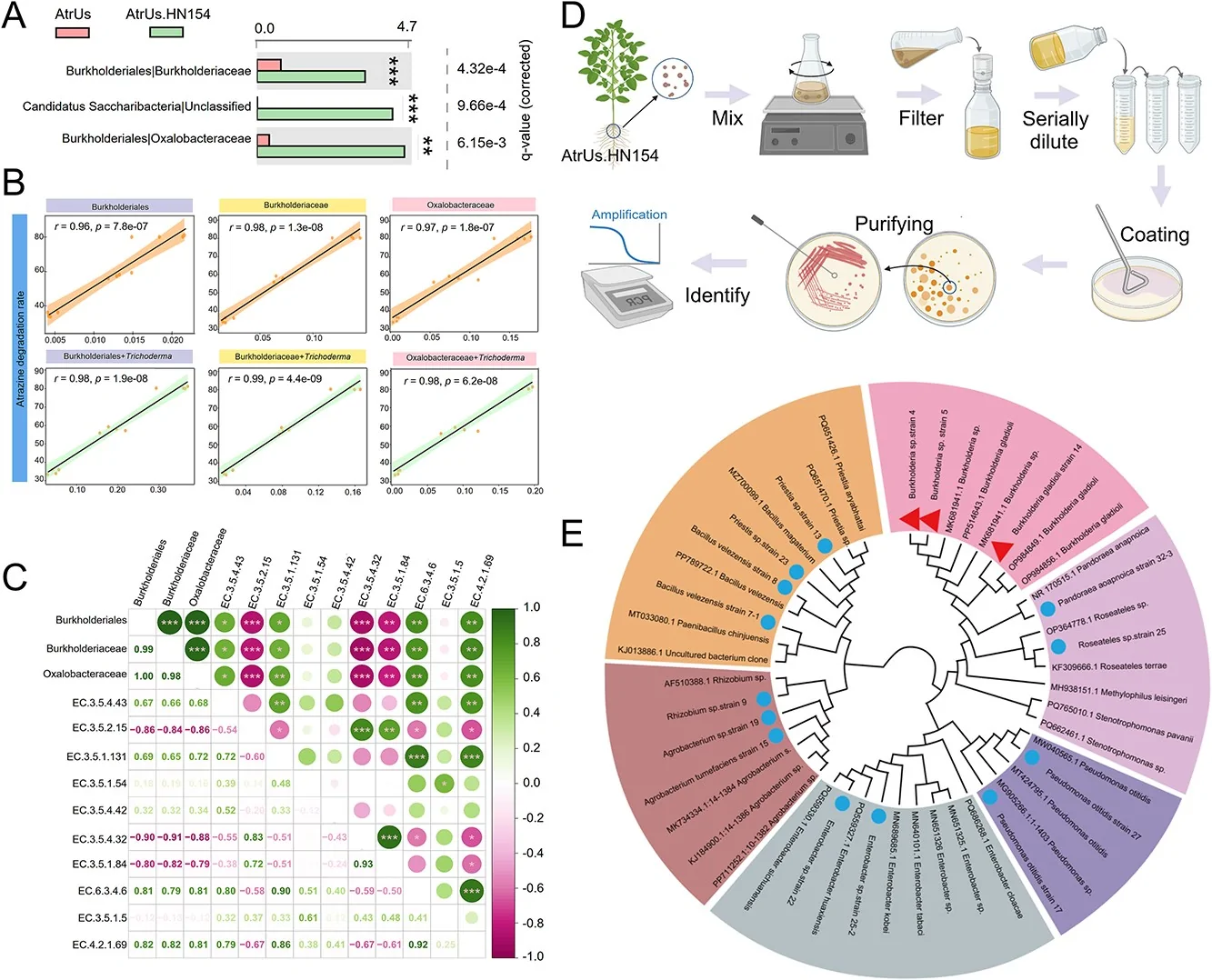
Identification of biomakers and isolation of atrazine-degrading bacteria. (A) Relative abundance and statistical significance of biomarker taxa (Burkholderiaceae, *Candidatus* Saccharibacteria, and *Burkholderiales* Oxalobacteraceae) samples with AtrUs versus AtrUs.HN154 treatments. Significance was assessed using Welch’s *t-*test with Storey false discovery rate (FDR) correction (*q* < 0.001). (B) Regression analysis of atrazine degradation rates against: relative abundances of *Burkholderiales*, Burkholderiaceae, Oxalobacteraceae, and combined relative abundances of *Burkholderiales,* Burkholderiaceae*,* Oxalobacteraceae with *T. lentiforme* HN154. (C) Heatmap of Spearman correlations between biomarker taxa (*Burkholderiales*, Burkholderiaceae, Oxalobacteraceae) and atrazine degraders' key enzymes. The color intensity scales with the absolute value of the Spearman correlation coefficient, where darker shades represent stronger correlations. The hue (red for positive, green for negative) denotes the direction of the correlation. This color mapping is presented in the legend panel on the right. (D) Schematic workflow for isolating culturable atrazine-degrading bacteria from the rhizosphere soil in AtrUs.HN154 treatment. (E) Phylogenetic tree of isolated bacterial strains was constructed on 16S rRNA gene sequences, constructed using the neighbor-joining method (bootstrap = 1000), isolated strains are identified as the *Burkholderiales* strain by a triangle, and another strain by a circle. Among them, AtrUs: Non-sterilized atrazine-contaminated soil (500 mg/kg). AtrUs.HN154: Non-sterilized atrazine-contaminated soil +50 ml HN154 spore suspension (10^8^ CFU/ml).

### Association between biomarkers and atrazine degradation in metagenomic annotation

We investigated the association between variations in the relative abundance of *Burkholderiales* (specifically its families Burkholderiaceae and Oxalobacteraceae) and differences in atrazine degradation efficiency across treatments. Regression analysis revealed strong correlations between atrazine degradation rates and the relative abundances of *Burkholderiales* (*P* = 7.8e-07, *r* = 0.96), Burkholderiaceae (*P* = 1.3e-08, *r* = 0.98), and Oxalobacteraceae (*P* = 1.8e-07, *r* = 0.97), as well as their combined with *Trichoderma* (Fig. 3B). Metagenomic annotation identified that the relative abundances of four atrazine-degradation key enzyme (EC 3.5.4.43 (*atzB*), EC 3.5.1.131 (*atzE*), EC 3.5.1.54 (*atzF*), EC 3.5.4.42 (*atzC*)) were positively correlated with these taxa in AtrSs.HN154, AtrUs, and AtrUs.HN154 treatments (Fig. 3C), suggesting their pivotal role in *Trichoderma*-assisted atrazine phytotoxicity.

### Isolation and characterization of atrazine-degrading bacteria

Screening bacterial strains that can grow in AtrUs rhizosphere on a medium containing 500 mg/l atrazine as the sole carbon/nitrogen source, 23 strains were finally selected (Fig. 3D). Following secondary screening, 19 strains exhibited measurable atrazine degradation activity (Fig. 3E), with strains Bur-4, Bur-5, and Bur-14 exhibiting the highest degradation efficiencies (29.4%, 26.3%, and 28.8%, respectively), the remaining isolates such as *Brevibacterium* sp. and *Enterobacter* sp. all showed weaker atrazine-degradation ability than the *Burkholderia* strains (Supplementary Table 5, Supplementary Fig. 6). 16S rRNA gene sequencing classified these isolates into three phyla (Pseudomonadota, Bacillota, Actinomycetota) and 11 genera. Bur-4, Bur-5, and Bur-14 were taxonomically assigned to *Burkholderia* (family Burkholderiaceae), aligning with the metagenomic findings (Supplementary Data 2).

### Functional validation of the *Trichoderma*–*Burkholderia* consortium

We constructed the *Trichoderma*–*Burkholderia* consortium HN154 + Burk, comprising *T. lentiforme* HN154 at 1 × 10^8^ CFU/ml and equal-volume mixture of three indigenous *Burkholderia* strains-Bur-4, Bur-5 and Bur-14-each also at 1 × 10^8^ CFU/ml, to evaluate its ability to degrade atrazine and alleviate phytotoxicity. Pot experiments showed that the HN154 + Burk consortium significantly enhanced atrazine degradation and reduced soybean phytotoxicity (Fig. 4A). Compared with the AtrSs control, AtrSs.HN154 increased plant height, fresh weight, and root length by 52.0%, 111.0%, and 61.0%, respectively. It also showed a 4.6-fold higher atrazine degradation rate, reaching 34.0% within 48 h (Fig. 4B).

**Figure 4:**
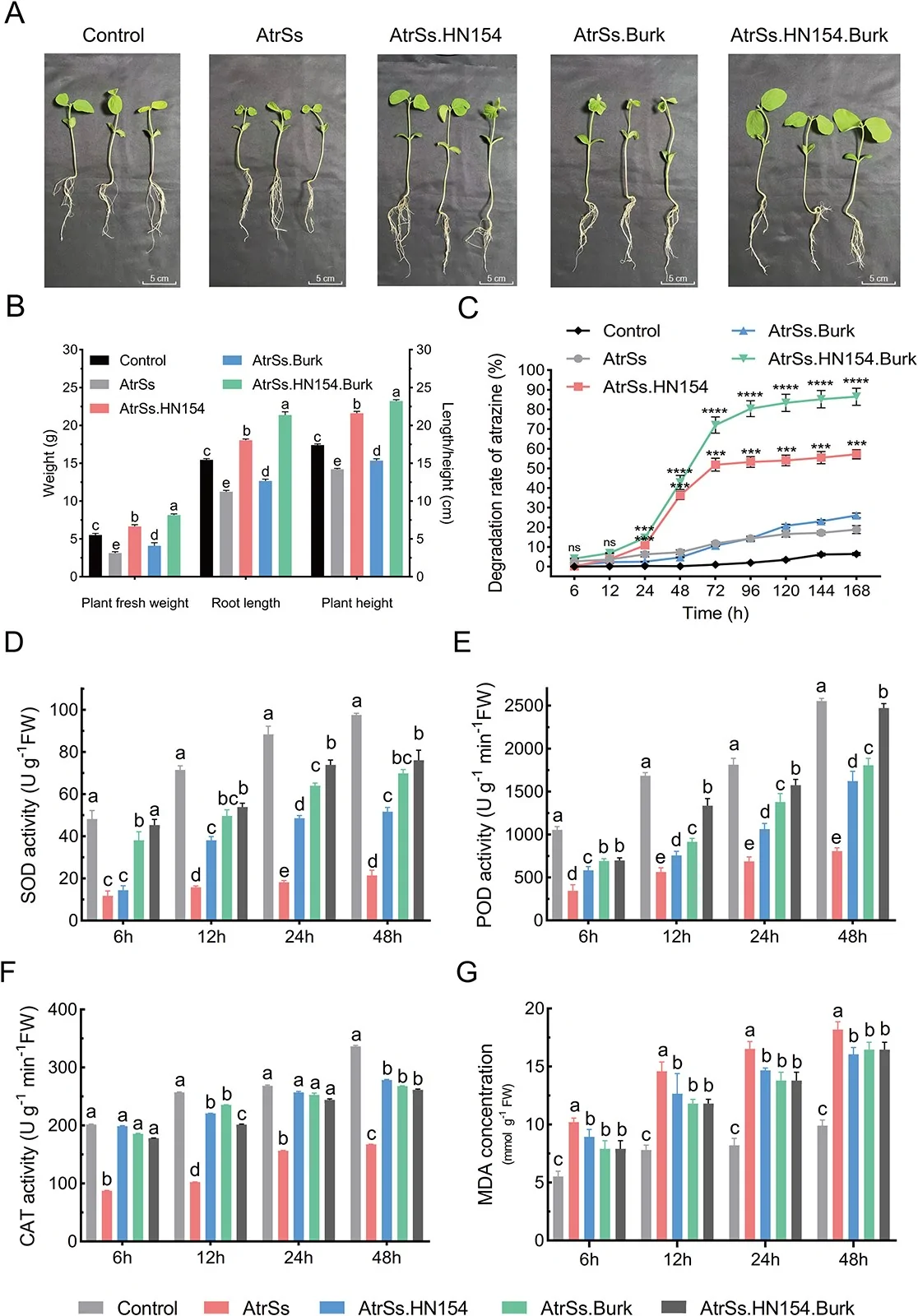
Effect of synthetic consortium HN154 + burk on plant growth and atrazine degradation in soil. (A) Soybean plants (14-day-old) under different treatments: Control, AtrSs, AtrSs.HN154, AtrSs.Burk, and AtrSs.HN154.Burk. (B) Fresh weight, plant height, and root length of soybean plants at 14 days post-treatment. (C) Atrazine degradation rates in soil at 6 h, 12 h, 24 h, 48 h, 72 h, 96 h, 120 h, 144 h, and 168 h (each treatment had three independent biological replicates. Each biological replicate was a mixture of rhizosphere soil from five soybean plants). (D-G) Impact of synthetic microbial consortium HN154 + burk on enzymatic activities (SOD, POD, and CAT) and the level of MDA in soybean roots. Among them, control: Sterile soil without atrazine. AtrSs: Sterile soil spiked with 500 mg/kg atrazine. AtrSs.HN154: AtrSs +50 ml HN154 suspension (1 × 10^8^ CFU/ml). AtrSs.Burk: AtrSs +50 ml bacterial community burk (1 × 10^8^ CFU/ml). AtrSs.HN154.Burk: AtrSs +50 ml consortium HN154 + burk (1 × 10^8^ cells/ml). Data are presented as mean ± SD from three independent experiments (n = 3 refers to three independent biological replicates). Statistical significance was analyzed by one-way ANOVA followed by Tukey’s HSD post-hoc test (*P <* .05). In panels D-G, different lowercase letters indicate significant differences among treatments. In panel C, asterisks denote significant differences between AtrSs.HN154.Burk or AtrSs.HN154 and AtrSs at a specific time point (^***^P < .001, ^****^P < .0001).

AtrSs.Burk showed comparatively lower efficacy relative to AtrSs.HN154 treatment. The synthetic consortium HN154 + Burk (AtrSs.HN154.Burk treatment) achieved optimal plant growth (plant height, fresh weight, and root length were 23.2 ± 0.19 cm, 8.2 ± 0.15 g, and 21.4 ± 0.44 cm, respectively) and rapid atrazine degradation (42.5% within 48 h, 86.3% within 168 h), surpassing all five treatments (*P <* .05) (Fig. 4C).

The phytotoxicity of atrazine results from the accumulation of excessive reactive oxygen species (ROS), while MDA serves as a key biomarker of membrane damage induced by oxidative stress. The synergistic actions of SOD, POD, and CAT maintain ROS homeostasis and protect cellular membrane integrity. Therefore, to delineate the phytotoxicity alleviation mechanisms of *T. lentiforme* HN154 and *Burkholderia* sp. strains, we measured the activities of MDA, SOD, POD, and CAT across five treatments: Control, AtrSs, AtrSs.HN154, AtrSs.Burk, and AtrSs.HN154.Burk. The AtrSs.HN154.Burk treatment showed 256% higher SOD and 211% higher POD activities versus AtrSs (*P* < .05), outperforming single-inoculant groups. Conversely, CAT activity was significantly elevated in AtrSs.HN154 and AtrSs.Burk compared to the consortium (*P* < .05). Furthermore, MDA content, peaked in AtrSs (18.2 ± 0.68 nmol g^−1^ FW) at 48 h post-treatment but decreased significantly by 10.94% in the consortium-treated (Fig. 4D–G). These findings mechanistically imply that the *Trichoderma*–*Burkholderia* consortium can also enhances atrazine tolerance through coordinated upregulation of antioxidant defense systems and concomitant mitigation of cellular oxidative injury.

### Inoculation with *T. lentiforme* HN154 alters the functional potential of the rhizosphere microbial community

To elucidate the regulatory role of *T. lentiforme* HN154 in soybean rhizosphere microbial community functions, we performed functional annotation of metagenomic data from ArtUs and ArtUs.HN154 treatments using the KEGG database. A total of 3 432 919 genes were identified, with 1 690 260 genes enriched in KEGG Level 3 pathways and 10 252 gene families mapped to KEGG Orthology (KO). The greater functional dispersion observed in the AtrUs.HN154 group compared to the AtrUs group (*P* < .05, Fig. 5A, Supplementary Figs 7 and 8) points to substantial community-level functional restructuring induced by *T. lentiforme* HN154.

**Figure 5:**
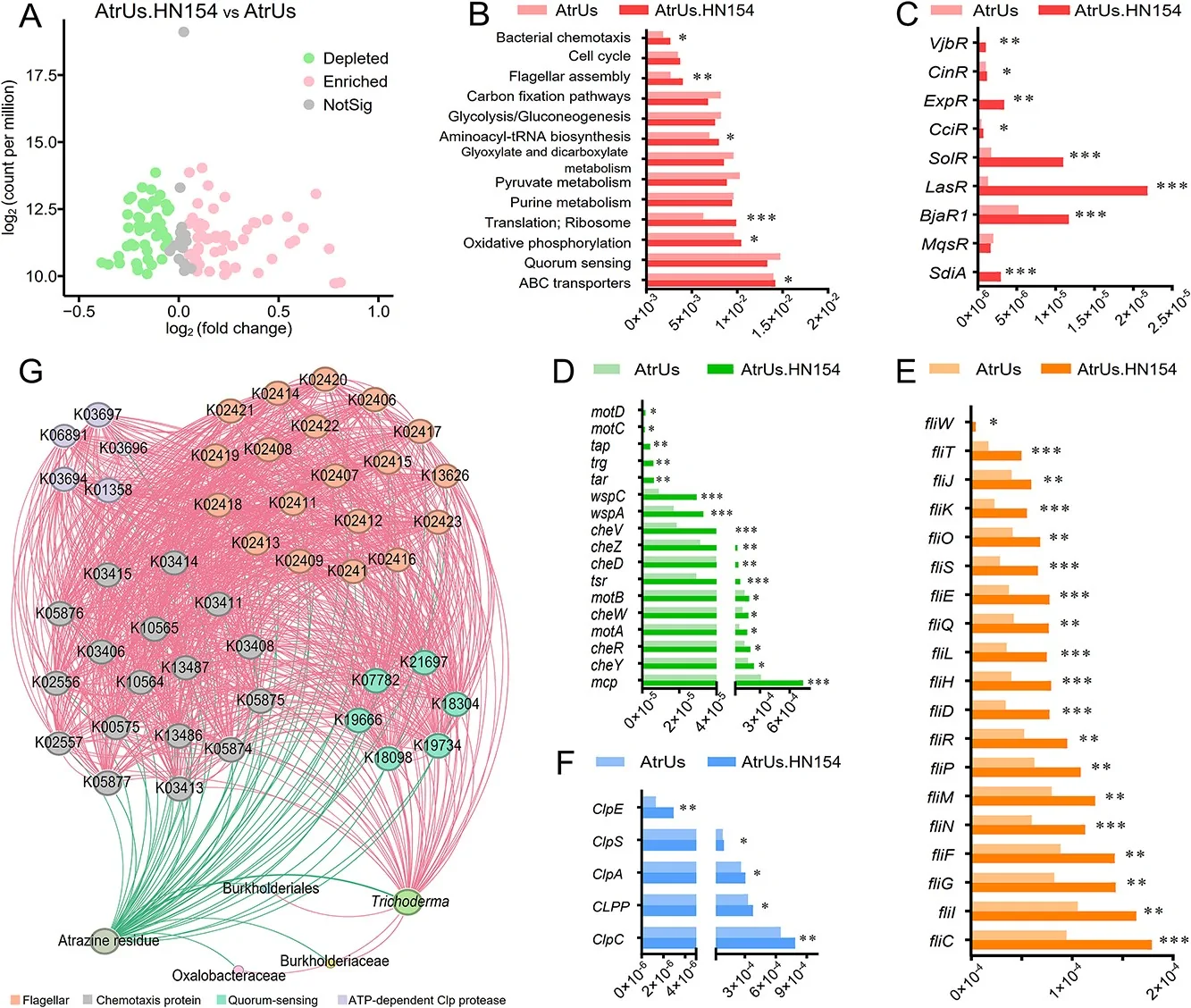
Regulation of functional genes in soybean rhizosphere microbial communities by *T. lentiforme* HN154 inoculation under atrazine stress. (A) Changes in Kyoto Encyclopedia of Genes and Genomes (KEGG) level 3 pathways. “Up” indicates upregulated expression, “Down” indicates downregulated expression, and “Nosig” denotes no significant changes. (B) Top 10 KEGG pathways ranked by relative abundance, along with the pathways related to bacterial chemotaxis, cell cycle, and flagellar assembly. (C-F) Significantly altered genes in specific functional pathways: quorum sensing (C), bacterial chemotaxis (D), flagellar assembly (E), and cell cycle (F). (G) Correlation network constructed based on *Trichoderma*, three key taxa (biomarker), 50 functional genes, and soil atrazine residues. Green and red edges represent negative and positive correlations, respectively, only significant (*P* < .05) and strong (|R| > 0.5) correlations are shown. Significant differences among treatments were analyzed by one-way ANOVA followed by Tukey’s HSD test (^*^P < .05, ^**^P < .01, ^***^P < .001). Among them, AtrUs: Non-sterilized atrazine-contaminated soil (500 mg/kg). AtrUs.HN154: Non-sterilized atrazine-contaminated soil +50 ml HN154 spore suspension (10^8^ CFU/ml).

Functional profiling identified 56 upregulated and 50 downregulated KEGG pathways between *T. lentiforme* HN154-treated and untreated samples. Pathways exhibiting significant differential expression included quorum sensing (QS), flagellar assembly, and bacterial chemotaxis (Fig. 5B). The KO-based analysis further highlighted upregulated genes associated with QS regulators (e.g. LuxR family), ATP-dependent Clp proteases (*K03696*, *K03694*), flagellar structural proteins (*K02411*, *K02415*), and chemotaxis proteins (*K02556*, *K03411*) (*P* < .05, Fig. 5C–F). The significant upregulation of specific genes indicates that *T. lentiforme* HN154 profoundly restructures the functional potential of the soil microbial community under atrazine stress (Supplementary Data 4).

### Degradation intermediates promote *Burkholderia* growth and motility

To elucidate the interaction between *T. lentiforme* HN154 activity and biomarker, we constructed a correlation network analysis integrating *T. lentiforme* HN154 abundance, biomarker taxa (Burkholderiales, Burkholderiaceae, Oxalobacteraceae), atrazine residues, and KO terms (*P* < .05, *R* > 0.5, Fig. 5G, Supplementary Fig. 9, Supplementary Data 5). *Trichoderma lentiforme* HN154 exhibited strong positive correlations with biomarkers and 50 orthologs enriched in QS (9 genes), flagellar assembly (19 genes), ATP-dependent proteolysis (5 genes), and chemotaxis (17 genes). All 50 orthologs had the highest relative abundance in the AtrUs.HN154 treatment, such as LuxR family transcriptional regulator (K19666, K18098, K18304), ATP-binding subunit (K03696, K03694, K03697), flagellar assembly protein (K02411, K13626), flagellar protein (K02415, K02418, K02413, K02423), and chemotaxis protein (K02556, K03411, K03414, K10564, K10565). The soil atrazine levels were negatively correlated with biomarker abundance and these functional orthologs, indicating that *T. lentiforme* HN154 enhances atrazine degradation in conjunction with a concurrent increase in the abundance of biomarker taxa and enrichment of genes for quorum sensing, flagellar assembly, ATP-dependent proteolysis, and chemotaxis. Therefore, we focused on the degradation intermediates generated during *T. lentiforme* HN154-mediated atrazine degradation via HPLC and validated their effects on the in vitro phenotypic traits such as growth dynamics and motility (chemotaxis, swimming, and swarming) of representative bacterial community Burk (Bur-4, Bur-5, and Bur-14) (Fig. 6). The chromatogram of atrazine degradation by *T. lentiforme* HN154 after 72 h revealed two peaks corresponding to the hydroxyatrazine and biuret standards. This suggests that these compounds formed as degradation intermediates (Fig. 6B). When cultured in ISM with atrazine as the sole carbon/nitrogen source, Burk exhibited limited growth (OD_600_ nm = 0.42). However, growth increased significantly by 124% (OD_600_ nm = 0.94) and 55% (OD_600_ nm = 0.65) when atrazine was substituted with biuret or hydroxyatrazine as the sole carbon/nitrogen source. Pre-inoculation of *T. lentiforme* HN154 in atrazine-containing ISM further enhanced *Burkholderia* growth (188% increase, OD_600_ nm = 1.21), indicating cross-feeding via intermediate metabolites (Fig. 6C–D). Furthermore, atrazine degradation intermediates (hydroxyatrazine and biuret) generated by *T. lentiforme* HN154, significantly enhanced chemotaxis, swarming and swimming capabilities of community Burk (*P* < .05, Fig. 6E). In contrast, neither atrazine (Atz) nor *T. lentiforme* HN154-secondary metabolites (HCB: ISM after 72 h HN154 culture and subsequent removed HN154) exhibited such stimulatory effects. These findings demonstrated a synergistic interaction between *T. lentiforme* HN154 and *Burkholderia* during atrazine degradation. *Trichoderma lentiforme* HN154 converts atrazine into bioavailable intermediates (hydroxyatrazine and biuret), which serve as more accessible carbon/nitrogen sources for *Burkholderia*, both of which are crucial properties for abundance enrichment in the rhizosphere and effective degradation.

**Figure 6:**
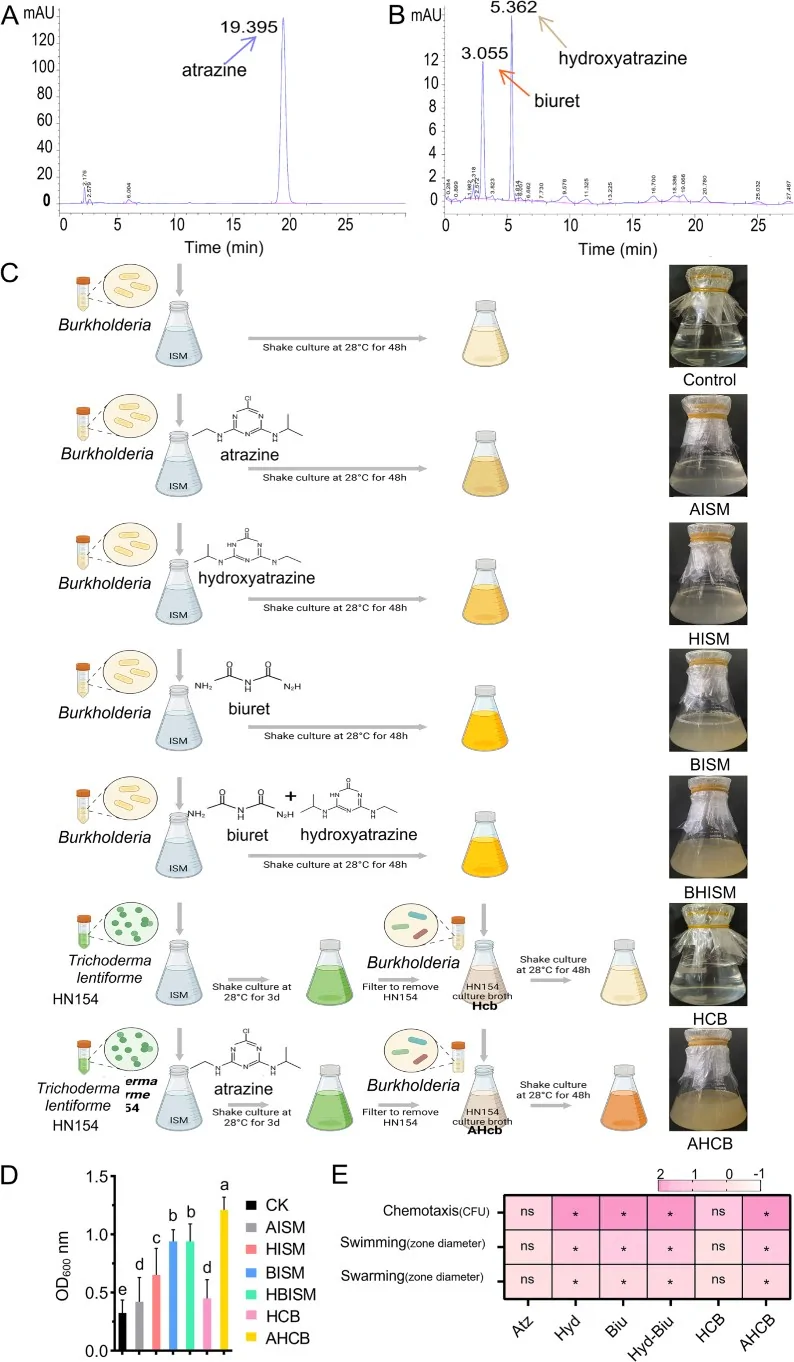
Intermediates of atrazine degradation by *T. lentiforme* HN154 and their effects on physiological responses of *Burkholderia* sp. (A, B) Representative LC chromatograms of the culture supernatant from *T. lentiforme* HN154 cultured with atrazine for 6 h (A) and 72 h (B). (C) Growth response of *Burkholderia* sp. when provided with different carbon/nitrogen sources, including atrazine, biuret, and hydroxyatrazine. Schematic diagram of experimental treatments (ISM, AISM, HISM, BISM, and BHISM). (D) Cell density (OD_600_ nm) values of *Burkholderia* sp. measured upon growth in the different conditions described in 6C. (E) Effect of degradation intermediates on physiological responses, swimming and swarming of *Burkholderia* sp. the color intensity corresponds to the magnitude of the chemotaxis index (*Rc*) and the swimming/swarming index (*Rs*). *Rc* was calculated as the ratio of CFUs in the test capillary to those in the control capillary, *Rc* ≥ 2 indicates a positive chemotactic response. *Rs* was calculated as (test diameter − control diameter)/control diameter, *Rs* ≥ 2 indicates enhanced motility. Data are presented as mean from three independent experiments (n = 3), * An *Rc* or *Rs* value of ≥2 is considered significant for this analysis. Abbreviations: ISM (inorganic salt medium). CK (*Burkholderia* sp. cultured in sterile ultrapure water). AISM (*Burkholderia* sp. cultured in ISM with 50 mg/l atrazine as the sole carbon and nitrogen source). HISM (*Burkholderia* sp. cultured in ISM with 50 mg/l hydroxyatrazine as the sole carbon and nitrogen source). BISM (*Burkholderia* sp. cultured in ISM with 50 mg/l biuret as the sole carbon and nitrogen source). BHISM (*Burkholderia* sp. cultured in ISM with 50 mg/l biuret and hydroxyatrazine as dual carbon/nitrogen sources). HCB (*Burkholderia* sp. cultured in cell-free broth prepared by growing HN154 in ISM for 72 h and then removing the fungal cells). AHCB (*Burkholderia* sp. cultured in HN154-culture broth-i.e, atrazine-based ISM precultured with HN154 for 72 h and then freed of the fungus, SMM based on atrazine after HN154 culture for 72 h and subsequent removal of HN154). Atz (*Burkholderia* sp. cultured in SMM supplemented with 50 mg/l atrazine). Hyd (*Burkholderi*a sp. cultured in SMM supplemented with 50 mg/l hydroxyatrazine). Biu (*Burkholderi*a sp. cultured in SMM supplemented with 50 mg/l biuret). Hyd-Biu (*Burkholderia* sp. cultured in SMM supplemented with both 50 mg/l hydroxyatrazine and biuret). Data are presented as mean ± SD from three independent experiments (n = 3). Statistical significance was determined by one-way ANOVA followed by Tukey’s HSD test (*P <* .05), with different lowercase letters indicating significant differences among treatments.

## Discussion

Atrazine contamination in agricultural soils remains a persistent global concern. Microbial remediation offers a cost-effective and environmentally sustainable strategy due to its lower ecological impact [58]. The inoculation of exogenous degrading microbes has proven effective in alleviating organic pollutant stress in sensitive plants. For instance, Tripathi *et al*. [59] demonstrated that the endophytic bacterium *Enterobacter* sp. CIMAP-A7 mitigates atrazine-induced phytotoxicity in *Andrographis paniculata*. Similarly, *Arthrobacter* sp. LY-1 [60] and *Paenarthrobacter ureafaciens* ZF1 [61] degraded 79.9% and 99.3% of soil atrazine (100 mg/kg) within seven and six days, respectively. In this study, under simulated field conditions, strain *T. lentiforme* HN154 degraded 80.3% of atrazine (500 mg/kg) in soil within 14 days, concurrently enhancing soybean growth and alleviating crop toxicity. The strain *T. lentiforme* HN154 exhibited superior atrazine tolerance compared to most reported atrazine-degrading microorganisms, and demonstrated higher degradation efficiency in non-sterilized soil than in sterilized soil, aligning with prior findings for *Acinetobacter* sp. strain YQJ-6, which demonstrated higher degradation rates in fresh soil (50 mg/kg) than in sterile soil [62]. This finding indicates that soil microbiota, which undergo structural and functional shifts upon exogenous microbial inoculation, play a key role in the biodegradation of atrazine and its intermediates, thereby facilitating the restoration of contaminated environments [63, 64]. We have also clarified that, under the sterilized + *Trichoderma* condition, the microbial community originated from surface-sterilized seeds and the introduced *Trichoderma* inoculum, as complete seed sterility is difficult to achieve. Regarding the robustness of our sequencing data, despite the constraint of having only four biological replicates, we implemented rigorous bioinformatic measures to mitigate compositionality bias. Consequently, the observed enrichment of atrazine-degrading taxa and the concomitant reduction in phytotoxicity could be attributed to the introduced consortium rather than to procedural artifacts. In our study, inoculation with *T. lentiforme* HN154 significantly altered the soil microbiota, with a notable enrichment of taxa, including Burkholderiales, Burkholderiaceae, and others. Members of Burkholderiaceae, particularly the genus *Burkholderia*, are ubiquitously distributed in soil, aquatic systems, and plant rhizospheres and are renowned for their capacity to degrade organic pollutants [65, 66]. For instance, *Burkholderia* sp. IDO3 degrades N-heterocyclic contaminants and indole derivatives [67], while *Burkholderia* sp. XTB-5 efficiently remediates phenol-contaminated soils [68]. Although direct evidence for atrazine degradation by *Burkholderia* sp. is currently lacking in the literature, we hypothesize that indigenous *Burkholderia* populations may enhance *T. lentiforme* HN154-driven atrazine degradation, based on their well-documented capacity to degrade a wide range of organic pollutants [69].

Using atrazine as the sole carbon/nitrogen source, we isolated 23 bacterial strains from the rhizosphere soil treated with AtrUs.HN154, 19 of which demonstrated atrazine degradation capability. The top three microbial degraders were identified as *Burkholderia* sp. (strains Bur-4, Bur-5, Bur-14), consistent with our metagenomic data. This reinforces the hypothesis that *Burkholderia* plays a pivotal role in complementing *T. lentiforme* HN154 to accelerate atrazine degradation in non-sterilized soils. Based on these findings, we constructed a synthetic microbial consortium using HN154 and Burk (consortium HN154 + Burk) and evaluated its atrazine degradation capacity (AtrSs.HN154 + Burk treatment). Although the degradation rates of the AtrSs.HN154.Burk and ArtUs.HN154 treatments did not differ significantly within the first 48 h, the degradation rate of the consortium was significantly higher than that of AtrSs.HN154 at 72–168 h. These results indicate that the addition of Burk facilitated atrazine degradation. Furthermore, the broader indigenous soil microbial community, which includes but is not limited to Burk, contributed to the enhanced degradation observed in non-sterile soil (ArtUs.HN154).

Atrazine disrupts Photosystem II (PSII) by blocking electron transport in photosynthesis, triggering oxidative imbalance and cellular necrosis [70]. This phytotoxicity stems from excessive accumulation of reactive oxygen species (ROS) accumulation [71]. The coordinated action of SOD, POD, and CAT maintains ROS homeostasis and preserves membrane integrity [72]. In this study, atrazine exposure (500 mg/kg soil, AtrSs group) significantly inhibited SOD, POD, and CAT activities in soybean roots. Nevertheless, inoculation with the consortium HN154 + Burk (AtrSs.HN154 + Burk treatment) restored these enzymatic activities beyond those observed in single-inoculation treatments (AtrSs.HN154, AtrSs.Burk) and the atrazine-stressed control (AtrSs). MDA, a critical biomarker of oxidative stress and lipid peroxidation-induced membrane damage, serves as a robust indicator for evaluating plant physiological status and cellular injury under environmental stress [73]. Similarly, the AtrSs treatment exhibited significant reductions in fresh biomass and elevated MDA accumulation in soybean roots, indicating that the atrazine concentration (500 mg/kg) exceeded the tolerance threshold of soybeans, thereby eliciting phytotoxic effects. The application of *T. lentiforme* HN154 and *Burkholderia* sp. (Burk) substantially attenuated MDA levels in soybean roots and restored plant growth. These findings suggest that *T. lentiforme* HN154 and *Burkholderia* sp. may mitigate atrazine-induced phytotoxicity by modulating ROS homeostasis, potentially through the upregulation of key antioxidant enzymes, including SOD, POD, and CAT. This enzymatic enhancement likely reduces ROS-driven lipid peroxidation, thereby lowering MDA accumulation and alleviating membrane damage.

Fungal probiotics can enhance host plant resilience by restructuring root-associated microbiomes [74]. However, the key drivers triggering this microbiota restructuring remain unclear. Bacterial quorum sensing (QS), a cell density-dependent communication system mediated by autoinducers such as *N*-acyl-homoserine lactones (AHLs), dynamically coordinates collective behaviors including biofilm formation and chemotaxis [75]. In the QS pathway, we observed significant upregulation of genes encoding transcriptional regulators *K19666 (solR)*, *K18304 (lasR)*, and *K18098 (bjaR1)* in AtrUs.HN154-treated soils. Significantly, *solR* (a *luxI/luxR* homolog) potentially modulates AHL signal synthesis, thereby regulating QS-driven chemotaxis and biofilm development in *Burkholderia* sp.

AtrUs.HN154 treatment exhibited the highest relative abundance of ATP-dependent Clp protease subunits *(K03696, K03694, K03697)* and flagellar assembly proteins (*K02411*, *K13626*). These components are critical for bacterial motility, stress adaptation, and biofilm dynamics [76]. Chemotaxis-associated proteins, including MotA (*K02556*), CheD (*K03411*), CheZ (*K03414*), MotC (*K10564*), and MotD (*K10565*), were also enriched, suggesting *T. lentiforme* HN154 enriched specific microbiota through motility-driven niche colonization [77]. Strikingly, soil atrazine levels showed a significantly negative correlation (*P* < .05) with biomarkers and 50 orthologs linked to QS (9 genes), flagellar assembly (19 genes), ATP-dependent proteolysis (5 genes), and chemotaxis (17 genes), all of them were abundant in AtrUs.HN154. This implied that *T. lentiforme* HN154-mediated atrazine degradation altered microbial chemotaxis systems, which could potentially have facilitated biomarker enrichment.

Microbial degradation of atrazine typically initiates via dechlorination or dealkylation (through deethylation or deisopropylation), yielding intermediates such as hydroxyatrazine (HA), desethylatrazine (DEA), and desisopropylatrazine (DIA) [78]. However, many isolated microorganisms from soil or aquatic environments fail to achieve complete mineralization of atrazine [79]. Most of these strains harbor only *trzN*, *atzB*, and *atzC* genes, converting atrazine to cyanuric acid via three enzymatic steps, such as *Pseudomonas* sp. ADP [80]. The certain intermediates exhibit higher toxicity than atrazine itself, raising concerns about causing problems with intermediate degradation product residues. Only a few microbial degraders carry *atzA*/*trzN*, *atzB*, *atzC*, *atzD*, *atzE*, and *atzF* genes to fully mineralize atrazine [81]. Metagenomic analysis reveals the relative abundance of genes encoding specific functions within the microbial community, indicating the genomic potential for atrazine degradation. The enrichment of *atzB* and *atzE* genes in the *Trichoderma*-inoculated treatment suggests the selective proliferation of atrazine-degrading populations. In this study, HPLC analysis revealed no detectable intermediates after 14 days of culturing *T. lentiforme* HN154 with atrazine as the sole carbon and nitrogen source, demonstrating that this strain could potentially achieve atrazine mineralization under the tested laboratory conditions. Burkholderiaceae abundance correlated positively with soil atrazine degradation genes (*atzB*, *atzE*, *P* < .05), and isolated *Burkholderia* strains (Bur-4, Bur-5, Bur-14) exhibited limited independent efficiency in atrazine degradation. We hypothesize that these strains may function as complementary mineralizers with indigenous microbiota. Transcriptomic or proteomic analysis would be required to confirm actual metabolic activity of these pathways.

Degradation intermediates, including hydroxyatrazine and biuret, were detected in 72 h *T. lentiforme* HN154 cultures (initial atrazine: 50 mg/l). Co-cultivation assays confirmed that *Burkholderia* strains (Bur-4, Bur-5, Bur-14) proliferated in *T. lentiforme* HN154-derived 3-day metabolic broth or ISM supplemented with hydroxyatrazine/biuret as sole C/N sources, indicative of metabolic cross-feeding on degradation byproducts. Hydroxyatrazine/biuret significantly enhanced *Burkholderia* motility (swimming and swarming) and elicited robust chemotactic responses (*P* < .01), suggesting that *T. lentiforme* HN154 may enrich these taxa via intermediate-driven chemoattractant gradients. Similarly, the fungal microbiome analysis revealed that phylogenetically related probiotic taxa were recruited by *Penicillium* sp. PQxj3 and reassembled the tobacco rhizosphere microbiome [82]. While improved motility, biofilm formation, and growth are recognized determinants of rhizosphere colonization [83], the molecular mechanisms underlying exogenous microbial degraders’ enrichment of indigenous microbiomes and their interactions remain poorly resolved.

Autoclaving inherently alters soil physicochemical properties [84], representing a potential limitation of this study. However, core comparisons were made within the same soil matrix (± *Trichoderma* in sterilized soil; ± *Trichoderma* in non-sterilized soil). This relative comparison design isolates the effect of *Trichoderma* addition by controlling for background soil variation [85]. Furthermore, the key finding regarding *Burkholderia* enrichment was derived from non-sterilized soil and is therefore unaffected by sterilization. Further validation using alternative sterilization methods (e.g. γ-irradiation) is warranted. In this study, atrazine was introduced into soil using acetone as a carrier. Although acetone was removed as thoroughly as possible through ventilation, its transient contact with soil may still exert potential effects that cannot be entirely ruled out [86]. Future studies should consider employing milder solvents (e.g. DMSO with appropriate controls) to better isolate the solvent effect from that of the target compound. Under the current experimental conditions, all acetone-treated samples (including controls) were processed consistently. Therefore, relative differences between treatments remain meaningful. The relatively small sample size may affect the strength of our correlation analyses. Future studies should therefore incorporate greater replication to improve the robustness of these findings. Deeper mechanistic insights into microbe-microbe interactions will advance our understanding of rhizosphere colonization and provide a theoretical basis for engineering microbial consortia that synergize with indigenous communities to enhance bioaugmentation efficiency.

## Conclusions

We employed the microbial degrader *T. lentiforme* HN154 in combination with metagenomic profiling, culturomic analyses, and synthetic community construction to elucidate how microbial community restructuring and functional gene abundance were regulated during atrazine degradation. Remediating atrazine-contaminated soil is essential for restoring ecological balance and ensuring agricultural sustainability. This study demonstrates that the exogenous fungus *T. lentiforme* HN154 enhances atrazine degradation and restructures the indigenous soil microbiome. The primary contribution of our work lies in the mechanistic elucidation of how HN154 releases atrazine metabolic intermediates that serve as growth substrates, thereby selectively enriching and synergizing with indigenous atrazine-degrading *Burkholderia* populations through chemotaxis and metabolic cross-feeding. This tripartite interaction among the fungal inoculant, pollutant metabolites, and resident microbiota forms a mutually beneficial microbial assemblage that accelerates pollutant breakdown, alleviates phytotoxicity (e.g. by reducing MDA content and upregulating activities of SOD, POD, and CAT), and reestablishes redox homeostasis. Our findings establish a conceptual and practical framework for microbiome-assisted bioaugmentation, highlighting the potential of synergistic microbial interactions to improve remediation efficiency and soil health. This approach bridges ecological principles with bioaugmentation strategies, advancing the sustainable restoration of contaminated agroecosystems (Fig. 7).

**Figure 7:**
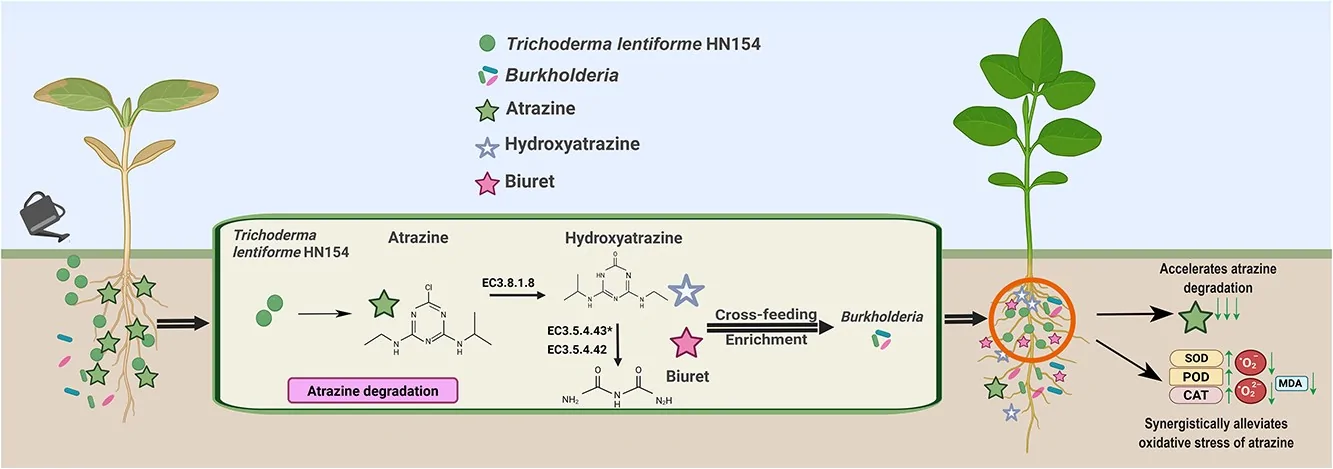
Conceptual model illustrating how *T. lentiforme* HN154 enhances atrazine biodegradation through cross-feeding *Burkholderia* with the intermediate metabolites from atrazine degradation. The exogenous fungal strain *T. lentiforme* HN154 colonizes the soybean rhizosphere, where it initiates atrazine degradation and produces intermediates including hydroxyatrazine and biuret. These intermediates function as chemoattractants and growth substrates, selectively enriching indigenous *Burkholderia* populations and promoting mutualistic interactions through chemotaxis and metabolic cross-feeding. The resulting *Trichoderma*–*Burkholderia* synthetic consortium synergistically accelerates atrazine degradation, alleviates plant oxidative stress (as indicated by reduced MDA content), and restores reactive oxygen species homeostasis by enhancing the activities of antioxidant enzymes such as SOD, POD, and CAT. Collectively, these processes contribute to the restoration of soil ecological health and improved crop resilience.

## Supplementary Material

Supplementary_material_wrag152

## Data Availability

Supporting data for all results presented in this paper are contained within the manuscript and supplementary materials. Metagenomic and gene sequencing data generated in this study are available in the National Genomics Data Center (NGDC) under accession number PRJCA042160. Soil metagenomic sequence samples list can be found in Supplementary Data 1–5. All other data generated and analyzed during this study can be requested from the corresponding author.
