# Supplementary material for: Trichoderma enriches Burkholderia via cross-feeding of degradation intermediates to enhance atrazine degradation and alleviate soybean phytotoxicity
Source: ISME J. 2026 Jun 29;20(1):wrag152. doi: 10.1093/ismejo/wrag152 (PMC13768756; doi:10.1093/ismejo/wrag152)
Supplement: Supplementary file 1 [file supplementary_material_wrag152.zip › Supplementary information.pdf]

Supplementary Figures

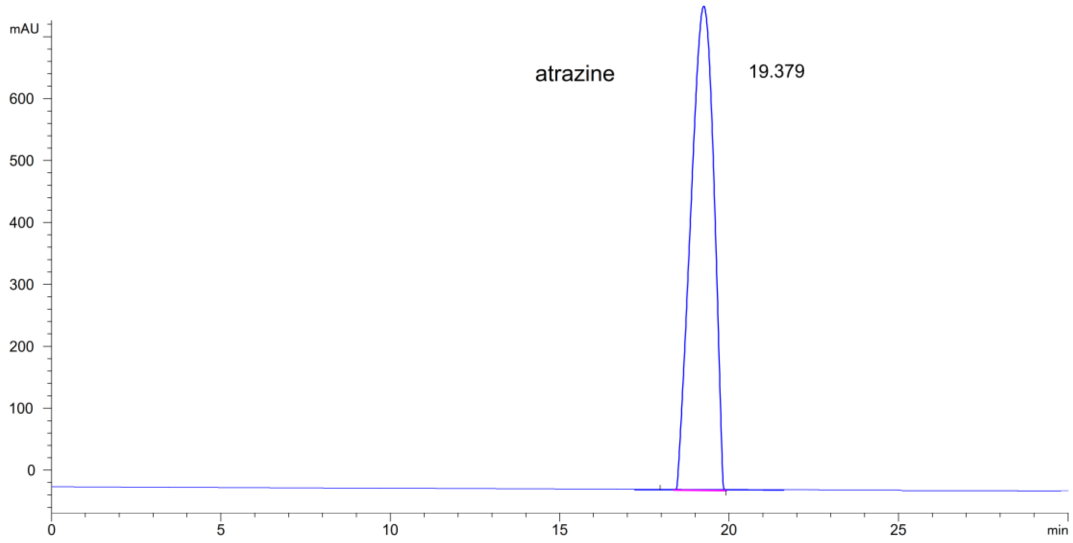

Supplementary Figure 1. HPLC chromatogram of standard samples of 1mg/L atrazine.

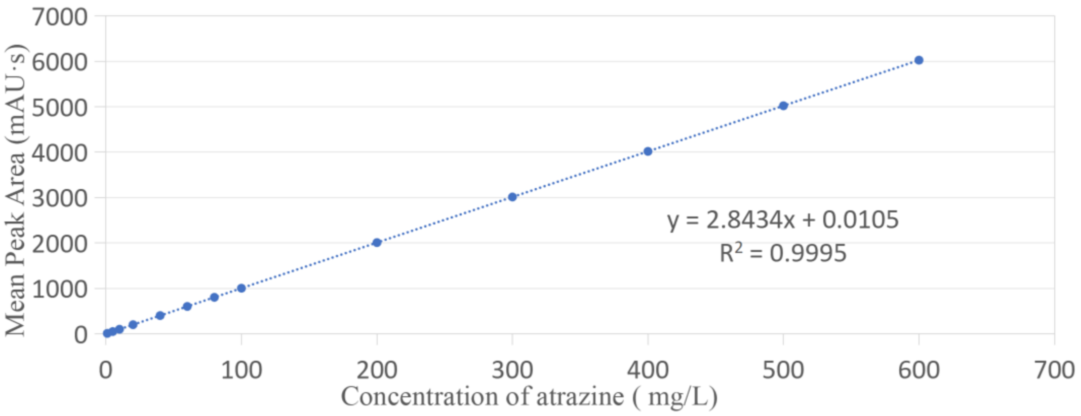

Supplementary Figure 2. Calibration curve illustrating the relationship between atrazine concentration (mg/L) and mean peak area (mAU·s) measured using high-performance liquid chromatography (HPLC). Data points represent experimental measurements derived from standard atrazine solutions, while the solid line corresponds to a linear regression fit (R^2 = 0.9995). No internal standard was used.

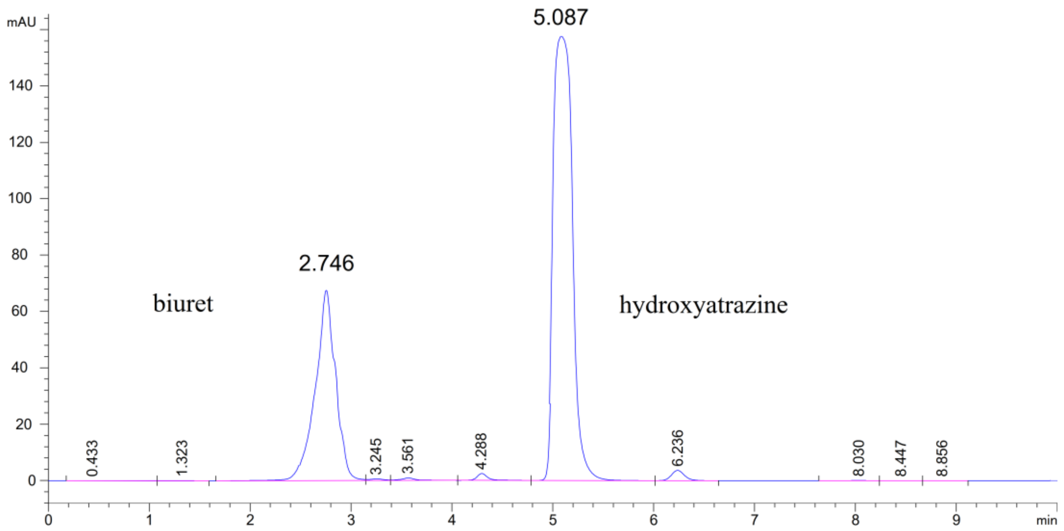

Supplementary Figure 3. HPLC chromatogram of standard samples of 1mg/L biuret and.

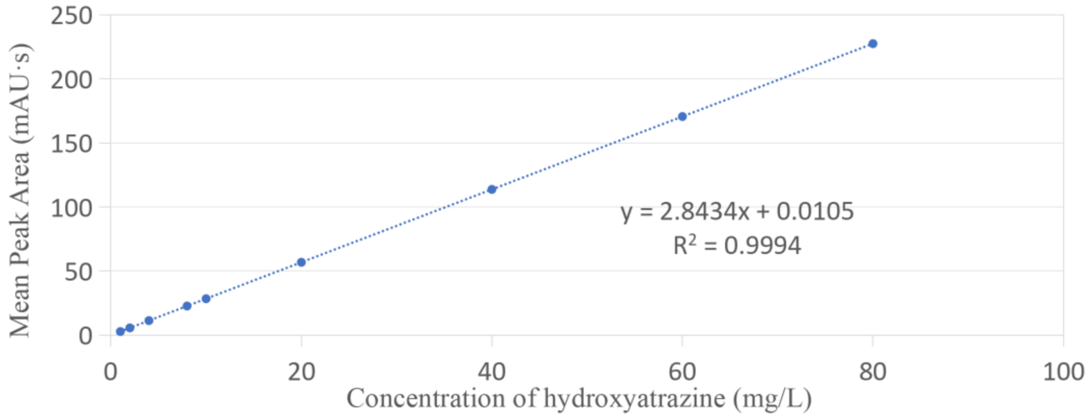

Supplementary Figure 4. Calibration curve illustrating the relationship between hydroxyatrazine concentration (mg/L) and mean peak area (mAU·s) measured using high - performance liquid chromatography (HPLC). Data points represent experimental measurements derived from standard hydroxyatrazine solutions, while the solid line corresponds to a linear regression fit ($R^2 = 0.9994$). No internal standard was used.

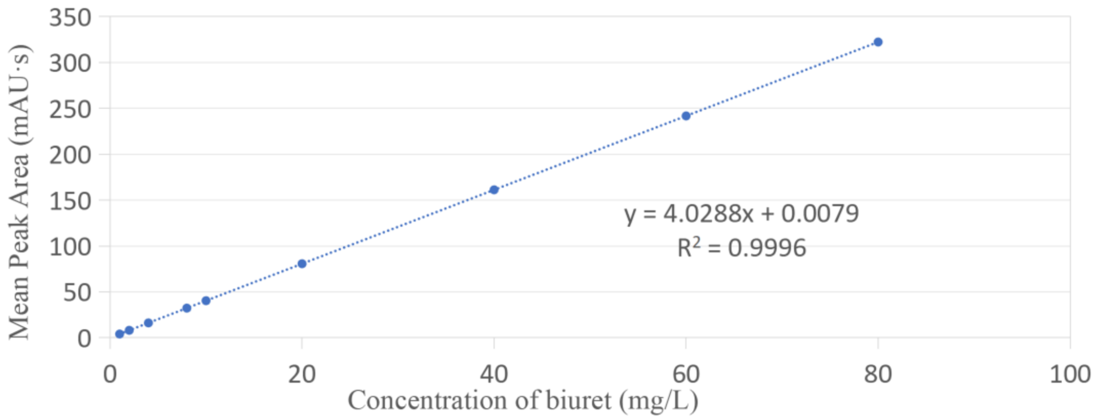

Supplementary Figure 5. Calibration curve illustrating the relationship between biuret concentration (mg/L) and mean peak area (mAU·s) measured using high-performance liquid chromatography (HPLC). Data points represent experimental measurements derived from standard biuret solutions, while the solid line corresponds to a linear regression fit (R^2 = 0.9996). No internal standard was used.

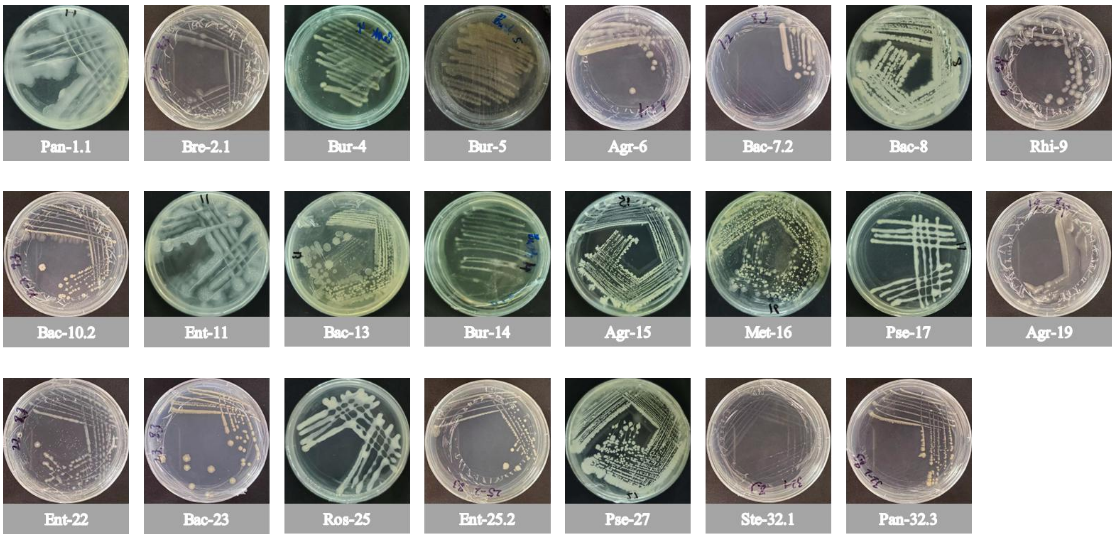

Supplementary Figure 6. Identification of 23 culturable atrazine - degrading bacteria.

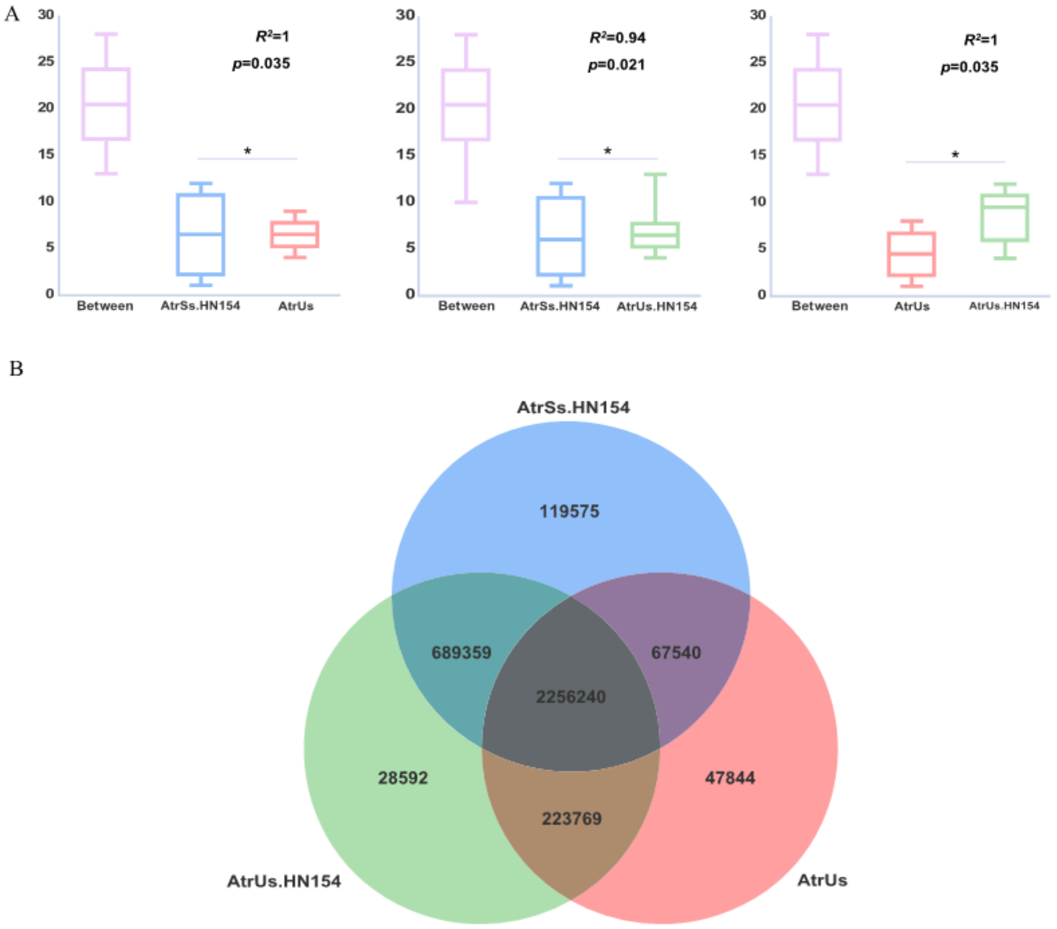

Supplementary Figure 7. Enrichment analysis of KEGG pathway between different treatment groups. **A**. Box plot comparison of KEGG Orthology (KO) abundance distribution between treatment groups. The color of the box represents different treatment groups, with the horizontal axis representing grouping conditions and the vertical axis representing KO relative abundance distribution ($P < 0.05$). **B**. Wayne plot analysis of shared and unique KO between different treatment groups. The overlapping area numbers represent the number of KOs shared between groups, while the non - overlapping area represents the number of KOs unique to each group.

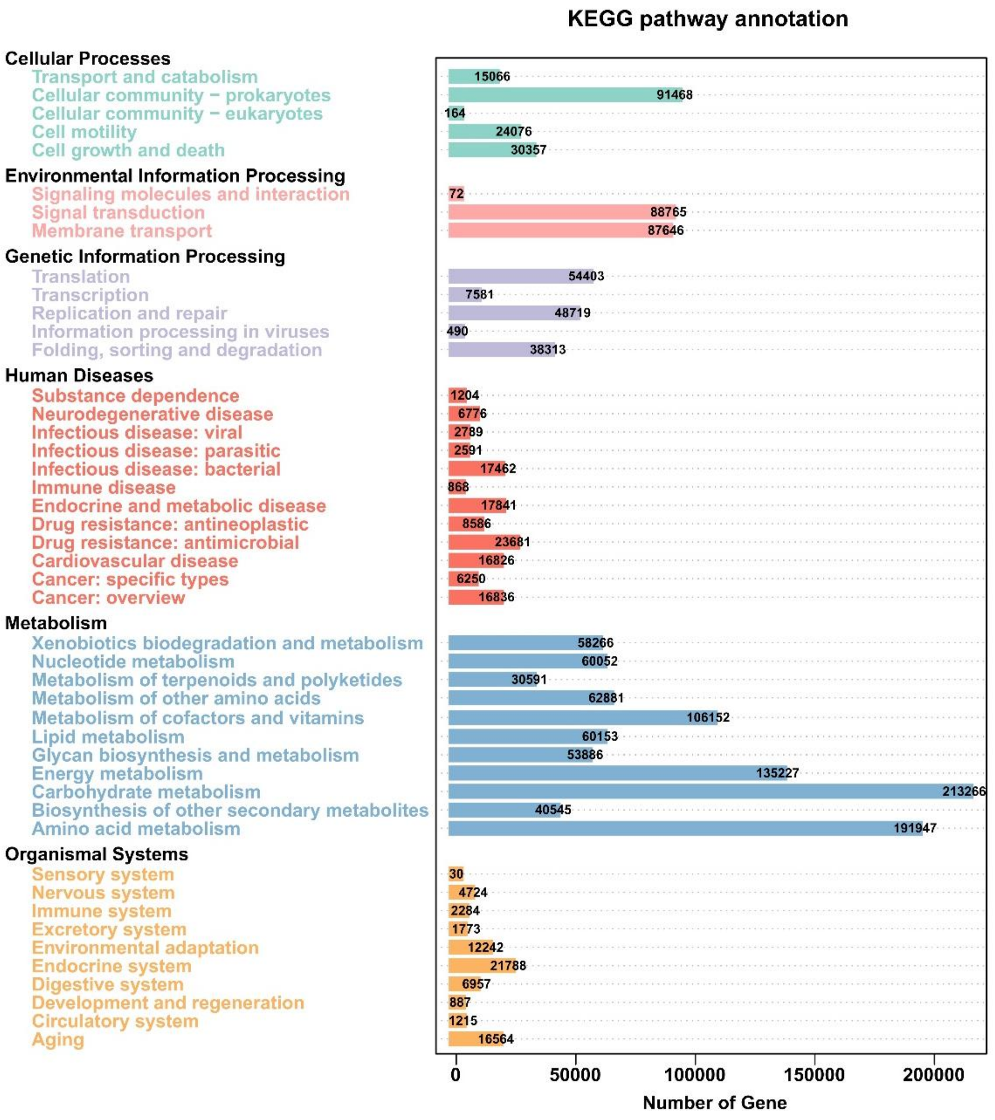

Supplementary Figure 8. The number of genes enriched in KEGG Level 3 pathways of AtrSs.HN154, AtrUs and AtrUs.HN154 treatments. Among them, AtrSs.HN154: Sterilized atrazine-contaminated soil + 50 mL *T. lentiforme* HN154 spore suspension (10^8 CFU/mL). AtrUs: Non - sterilized atrazine - contaminated soil (500 mg/kg). AtrUs.HN154: Non - sterilized atrazine - contaminated soil + 50 mL *T. lentiforme* HN154 spore suspension (10^8 CFU/mL).

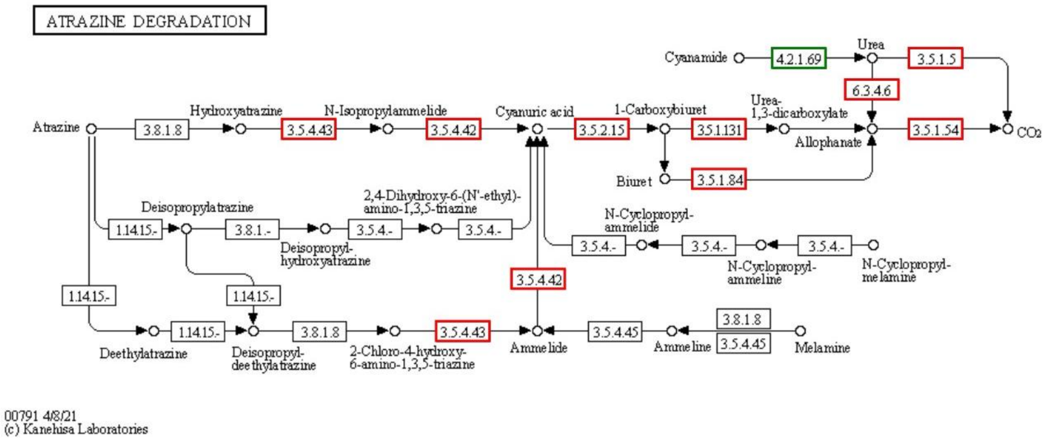

Supplementary Figure 9. The microbial atrazine degradation pathway and annotated enzymatic reactions in the AtrUs and AtrUs.HN154 treatments. Enzymatic reactions common to both treatments are depicted in red, while those unique to the AtrUs.HN154 treatment are highlighted in green. Among them, AtrUs: Non - sterilized atrazine-contaminated soil (500 mg/kg). AtrUs.HN154: Non-sterilized atrazine - contaminated soil + 50 mL *T. lentiforme* HN154 spore suspension (10^8 CFU/mL).

Supplementary Tables

Supplementary Table 1. Chromatographic parameters for target compounds.

Analyte	Flow (mL/min)	λ (nm)	tR (min)	LOD (μg/ L S/N = 3)	Percent Recovery
atrazine	1.0	220	24.162	1.8	96(n=5)
hydroxyatrazine	1.0	254	5.087	2.5	95(n=5)
biuret	1.0	200	2.746	3.2	97(n=5)

Supplementary Table 2. PERMANOVA results (Bray - Curtis distance, 9,999 permutations) among atrazine - degrading strain.

Source	Df	SumOfSqs	F.Model	R^2	Pr(>F)
Group	2	0.4568	38.9	0.865	0.001
Residual	9	0.0528	—	0.135	—
Total	11	0.5096	—	1.000	—

Non - normal (Shapiro-Wilk $P < 0.05$), Spearman correlation used.

Supplementary Table 3. LEfSe - identified biomarkers (LDA > 4.0, P < 0.05) distinguishing AtrUs vs AtrUs.HN154 (non - sterilized soil).

Taxonomic rank	Taxon	Log_{10} Average Abundance	LDA	KW P-value
Order	Burkholderiales	4.908292560996221	4.620394181251675	0.020921335337793993
Class	Betaproteobacteria	4.9538212607461505	4.58651667452537	0.020921335337793993
Genus	*Massilia*	4.6496507029947445	4.345443385004109	0.020921335337793993
Family	Oxalobacteraceae	4.669348915899212	4.344454110366473	0.020921335337793993
Phylum	Candidatus Saccharibacteria	4.5431723277777065	4.255467306089486	0.020921335337793993
Family	Burkholderiaceae	4.53479582441266	4.231723108516005	0.020921335337793993
Genus	*Pandoraea*	4.53479582441266	4.197293918291906	0.020921335337793993

Supplementary Table 4. Relative abundance and statistical significance of biomarker taxa (Burkholderiaceae, *Candidatus Saccharibacteria*, and Burkholderiales Oxalobacteraceae) samples with AtrUs versus AtrUs.HN154 treatment.

Family	AtrUs: mean rel. freq. (%)	AtrUs: std. dev. (%)	AtrUs.HN154: mean rel. freq. (%)	AtrUs.HN154: std. dev. (%)	*P*-values	*P*-values (corrected)	Difference between means	99.0% lower CI	99.0% upper CI
Burkholderiales Burkholderiaceae	0.763294499	0.118751175	3.445620548	0.225191454	1.98E-05	0.000432458	-2.68232605	-3.306427956	-2.058224143
Candidatus Saccharibacteria Unclassified	0.01056806	0.003923065	4.320370006	0.392610594	0.000317342	0.000965692	-4.309801946	-5.633587281	-2.986016611
Burkholderiales Oxalobacteraceae	0.391739309	0.063050843	4.699123608	1.185606104	0.008003403	0.006153487	-4.307384299	-8.288949511	-0.325819088

Supplementary Table 5. Sequencing results and atrazine degradation rates of 23 culturable bacterial strains.

Serial number	Strain number	Closest genus	NCBI GenBank Accession No.	Maximum similarity (%)	Degradation rate of herbicide (%)
1	Pan-1.1	*Pandoraea* sp.	OQ024845.1	100.00	13.68±1.03 b
2	Bre-2.1	*Brevibacterium* sp.	PQ359582.1	99.78	16.33±0.36 ab
3	Bur-4	*Burkholderia* sp.	MK681941.1	100.00	29.40±1.36 a
4	Bur-5	*Burkholderia* sp.	PP514643.1	100.00	26.30±0.21 a
5	Agr-6	*Agrobacterium* sp.	MT415399.1	100.00	12.03±1.69 b
6	Bac-7.1	*Bacillus* sp.	MT033080.1	100.00	14.79±0.66 ab
7	Bac-8	*Bacillus* sp.	PP789722.1	100.00	11.47±1.65 b
8	Rhi-9	*Rhizobium* sp.	AF510388.1	100.00	9.72±0.67 c
9	Bac-10.2	*Bacillus* sp.	OR263040.1	100.00	14.66±1.58 b
10	Ent-11	*Enterobacter* sp.	MN589806.1	100.00	10.34±0.55 c
11	Bac-13	*Bacillus* sp.	PQ651470.1	100.00	5.36±0.65 d
12	Bur-14	*Burkholderia* sp.	OP984849.1	100.00	28.83±0.79 a
13	Agr-15	*Agrobacterium* sp.	PP711252.1	100.00	11.12±1.34 ab
14	Met-16	*Methylophilus* sp.	KX022799.1	100.00	12.36±0.31 ab
15	Pse-17	*Pseudomonas* sp.	MG905266.1	100.00	10.75±0.65 c
16	Agr-19	*Agrobacterium* sp.	KJ148900.1	100.00	10.21±0.48 c
17	Ent-22	*Enterobacter* sp.	PQ559330.1	100.00	6.35±0.16 d
18	Bac-23	*Bacillus* sp.	MZ700099.1	100.00	15.44±0.75 ab
19	Ros-25	*Roseateles* sp.	KF309666.1	100.00	6.2±0.66 d
20	Ent-25.2	*Enterobacter* sp.	PQ559327.1	100.00	16.54±0.48 ab
21	Pse-27	*Pseudomonas* sp.	MW040565.1	100.00	14.3±0.64 ab
22	Ste-32.1	*Stenotrophomonas* sp.	MG905311.1	100.00	11.63±0.35 c
23	Pan-32.3	*Pandoraea* sp.	NR170515.1	100.00	10.63±0.44 c
